# PCLAF induces bone marrow adipocyte senescence and contributes to skeletal aging

**DOI:** 10.1038/s41413-024-00337-5

**Published:** 2024-07-04

**Authors:** Lingqi Xie, Yalun Cheng, Biao Hu, Xin Chen, Yuze An, Zhuying Xia, Guangping Cai, Changjun Li, Hui Peng

**Affiliations:** 1 Department of Endocrinology, Endocrinology Research Center, Xiangya Hospital of Central South University, Changsha, Hunan 410008 China; 2 National Clinical Research Center for Geriatric Disorders, Xiangya Hospital, Changsha, Hunan 410008 China; 3 Key Laboratory of Organ Injury, Aging and Regenerative Medicine of Hunan Province, Changsha, Hunan 410008 China

**Keywords:** Metabolic bone disease, Metabolic disorders

## Abstract

Bone marrow adipocytes (BMAds) affect bone homeostasis, but the mechanism remains unclear. Here, we showed that exercise inhibited PCNA clamp-associated factor (PCLAF) secretion from the bone marrow macrophages to inhibit BMAds senescence and thus alleviated skeletal aging. The genetic deletion of PCLAF in macrophages inhibited BMAds senescence and delayed skeletal aging. In contrast, the transplantation of PCLAF-mediated senescent BMAds into the bone marrow of healthy mice suppressed bone turnover. Mechanistically, PCLAF bound to the ADGRL2 receptor to inhibit AKT/mTOR signaling that triggered BMAds senescence and subsequently spread senescence among osteogenic and osteoclastic cells. Of note, we developed a PCLAF-neutralizing antibody and showed its therapeutic effects on skeletal health in old mice. Together, these findings identify PCLAF as an inducer of BMAds senescence and provide a promising way to treat age-related osteoporosis.

## Introduction

Skeletal tissue undergoes dynamic remodeling throughout the life course, which is coordinated by bone formation and bone resorption.^1–3^ However, aging disrupts the bone dynamic equilibrium. Thus, compared to high bone remodeling of postmenopausal osteoporosis, low bone remodeling was observed in senile osteoporosis characterized by decreased bone formation and resorption.^4–7^

The accumulated BMAds during aging impaired bone homeostasis, which increased the risks of fracture and osteoporosis.^8–10^ Previous studies majorly focused on the switched cell fate of bone marrow mesenchymal stem cells (BMSCs) between osteoblasts and adipocytes.^11,12^ Other opinions claimed that the BMAds accumulation induced lipotoxicity in the bone marrow and thus caused bone loss.^13–15^ Recently, BMAds senescence was firstly reported to damage osteogenesis and angiogenesis which was involved in the glucocorticoid-induced bone deterioration.^16,17^ Yet, the aspects of BMAds senescence on age-related bone loss are rarely investigated.

Exercise can improve bone health and reduce the risk of bone loss.^18–20^ To date, the benefits of mechanical loading on the skeleton accounted for the release of exercise-stimulated myohormone and the suppression of senescence-associated secretory phenotype (SASPs)-related factors.^21–23^ In addition, bone marrow fat has been reported to be critical for exercise-stimulated bone formation.^18,24–26^ Our previous study found that exercise stimulated the secretion of reticulocalbin-2 from bone marrow macrophages, which promoted the lipolysis of BMAds and provided energy substrates to maintain skeletal homeostasis.^24^ However, it is unclear if exercise regulates BMAds senescence to alleviate skeletal aging.

In our study, we identified a bone marrow macrophage (BMM)-derived senescent inducer, proliferating cell nuclear antigen clamp-associated factor (PCLAF), which promoted BMAds senescence and bone loss. Mechanistically, PCLAF binding to the ADGRL2 receptor, accelerated BMAds senescence and promoted bone loss. Notably, we have developed a PCLAF-neutralizing antibody and demonstrated its therapeutic potential in improving bone health in old mice.

## Results

### Exercise alleviates BMAds senescence and bone aging

**Fig. 1:**
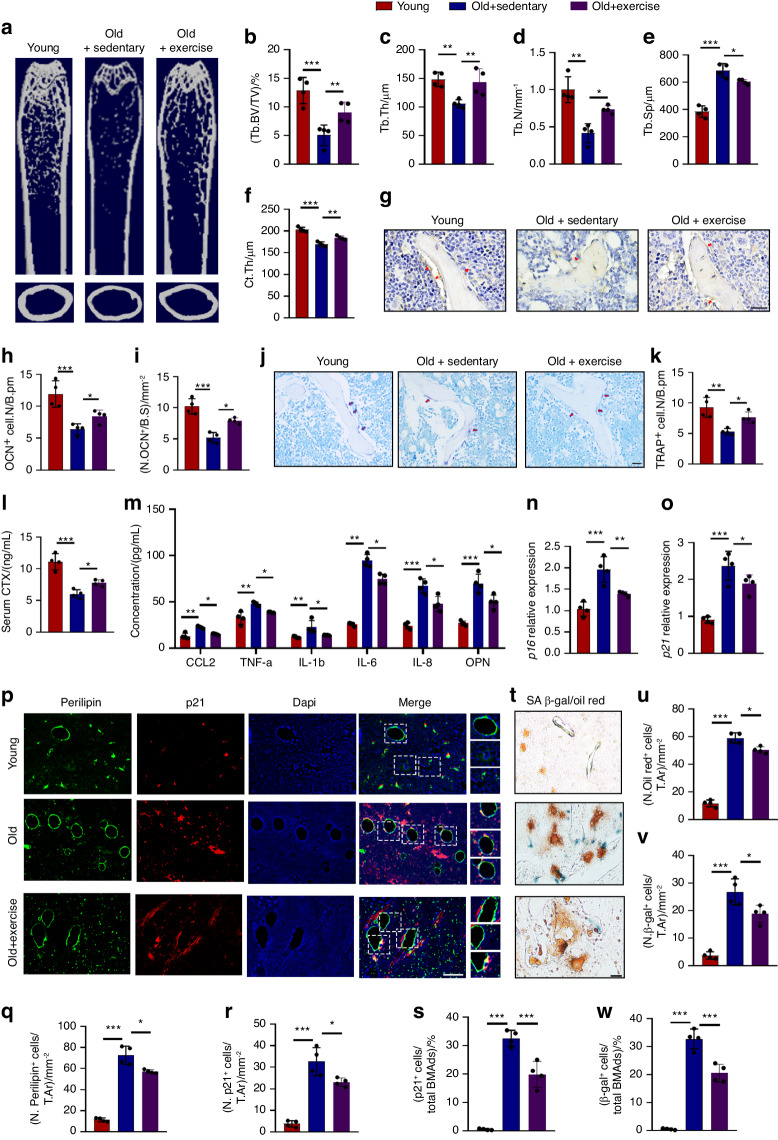
Exercise alleviates BMAds senescence and bone aging. **a** Representative image of Micro-CT of young (3-month), old (15-month) sedentary and exercise old mice (*n* = 4). **b**–**f** Quantitative analysis of trabecular bone volume (Tb.BV/TV, **b**) trabecular thickness (Tb.Th, **c**), trabecular bone number (Tb.N, **d**), trabecular separation (Tb.Sp, **e**), cortical bone thickness (Ct.Th, **f**). (n = 4). **g**–**i** Representative images of osteocalcin (OCN) staining (**g**) and quantification (**h**, **i**) of number of OCN^+^ cells (red arrows) in femurs (scale bar, 50 μm; *n* = 4). **j**, **k** Representative images of TRAP staining (**j**) and quantification (**k**) of number of TRAP^+^ cells (red arrows) in femurs (scale bar, 50 μm; *n* = 4). **l** Serum CTX level of young (3-month), old (15-month) sedentary and exercise mice (*n* = 4). **m** Quantitative analysis of SASPs (CCL2, TNF-α, IL-1b, IL-6, IL-8, OPN) in bone marrow supernatant of young mice, old sedentary and exercise old mice. **n**, **o** QPCR analysis of *p16* (**n**) and *p21* (**o**) in BMAds of young, old sedentary and exercise old mice. **p** Representative images of immunofluorescence staining of p21 and perilipin (scale bar, 50 μm; *n* = 4). **q**–**s** Quantification of the number of perilipin^+^ cells per mm^2^ tissue area (N. Perilipin^+^ cells, **q**), the number of p21^+^ cells per mm^2^ tissue area (N. p21^+^ cells, **r**), and the percentage of p21^+^ cells in total perilipin^+^ cell population ((p21^+^ cells/total BMAds)/%, **s**). **t** Representative images of SA β-gal and Oil Red O staining (scale bar, 50 μm; *n* = 4). **u**–**w** Quantification of the numbers of Oil Red O^+^ cells per mm^2^ tissue area (**u**), SA β-gal^+^ cells per mm^2^ tissue area (**v**), and the percentage of SA β-gal^+^ cells in total oil red^+^ BMAds respectively (**w**). Data are shown as the mean ± SD. *P < 0.05, **P < 0.0,. ***P < 0.001 by two-way ANOVA (**b**–**f**, **h**, **i**, **k**–**o**, **q**–**s**, **u**–**w**)

Exercise is beneficial to bone health both in young mice and old mice.^18,27^ To figure out the mechanisms, we firstly collected the femurs of 3- and 15-month-old mice that either lived on the ground (free access to nesting material and food) or running (10 m/min, 30 min/day for 21 days) lifestyle. Micro-CT showed that sedentary old mice showed a lower trabecular volume, thickness, number, and cortical bone thickness but higher trabecular separation compared to the young mice. However, old mice with running intervention showed relatively high bone mass compared to age-matched sedentary mice (Fig. 1a–f). Moreover, old mice showed fewer osteocalcin^+^ osteoblastic and TRAP^+^ osteoclastic cells compared to young mice, and this decrease was partially blocked by exercise intervention (Fig. 1g–k). Consistent with TRAP staining, the C-terminal telopeptide of type I collagen (CTX) showed a lower level in old mice with running intervention than in old mice without running intervention (Fig. 1l). Together, these results indicated that exercise could alleviate skeletal aging.

Cellular senescence occurs during aging and thus secreting SASPs in the bone marrow.^28,29^ To figure out whether exercise alleviated cellular senescence, we detected a list of well-established SASPs in bone marrow supernatants and found a reduced level of these SASPs after exercise (Fig. 1m). Peripheral white adipose tissue in mice showed age-related gene expression changes earlier than other organs,^30^ indicating that adipocytes may be precursors of the aging process. Considering the significant proportion of bone marrow adipose tissue (BMAT) in the bone marrow, especially during aging, we explored whether BMAT senescence contributed to skeletal aging. Surprisingly, we found an increased expression level of *p16* and *p21* in BMAds of sedentary old mice while it decreased after running (Fig. 1n, o). Sedentary old mice also exhibited more senescent adipocytes compared to young mice as indicated by a greater percentage of p21^+^ perilipin^+^ cells of the total perilipin^+^ cells, which were partially reversed in exercised mice (Fig. 1p–s). Additionally, Oil red O and SA β-gal staining of the bone tissue sections showed a consistent result as senescent β-gal^+^ BMAds remained higher in sedentary old mice relative to young mice whereas exercise counteracted the BMAds senescence (Fig. 1t–w). Together, these findings suggest that exercise alleviates BMAds senescence and delays bone aging.

### Exercise inhibits the secretion of PCLAF from bone marrow macrophages

**Fig. 2:**
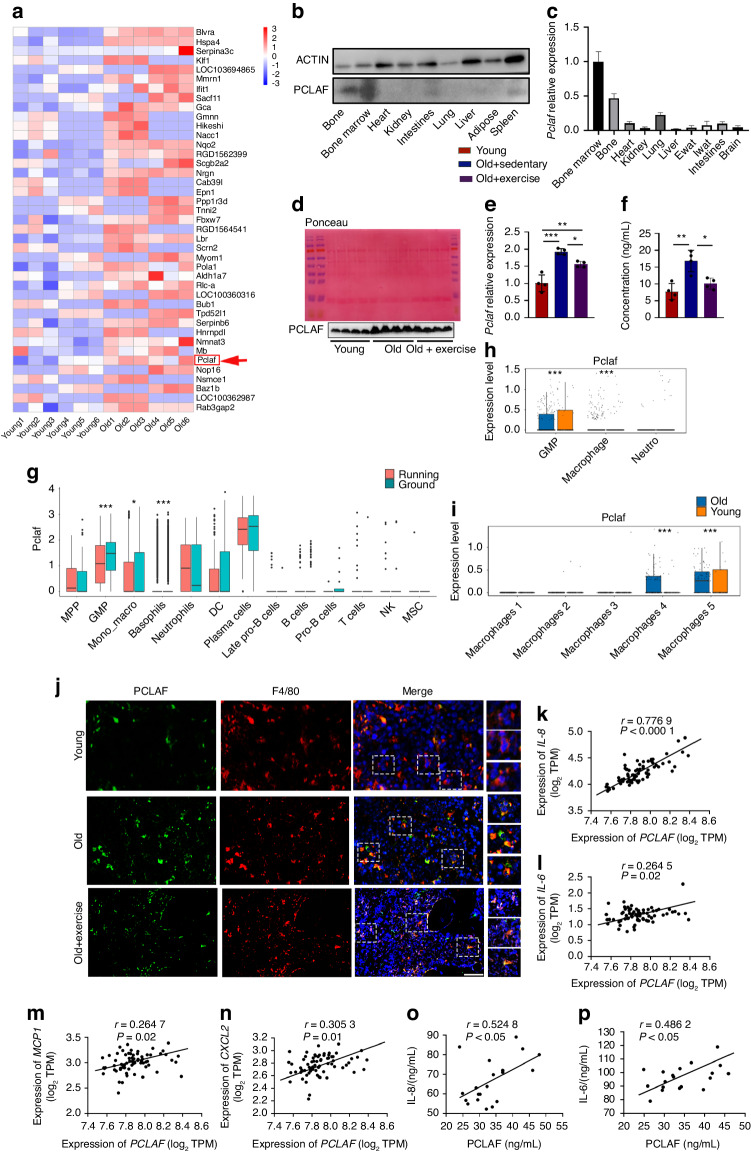
Exercise inhibits the secretion of PCLAF from bone marrow macrophages. **a** Heatmap of Top40 upregulated factors in the bone marrow supernatant of young (3-month) and old (24-month) male rats (*n* = 6). **b** Representative western blot image of PCLAF expression in various tissues. **c** Quantification of western blot of PCLAF expression in various tissues. **d** Representative Ponceau S staining and western blot of PCLAF expression of bone marrow supernatant from young, old sedentary or exercise old mice. **e** Quantification of western blot of PCLAF expression of bone marrow supernatant. **f** Quantitative analysis of PCLAF in bone marrow supernatant (*n* = 4). **g** Box plots of log-transformed *Pclaf* expression in different cell clusters of bone marrow (ground versus running mice). Previous published scRNA-seq data (GEO: GSE202710) were re-analyzed here. **h** Box plots of log-transformed *Pclaf* expression in different cell clusters of bone marrow (young versus old rats). Previous published scRNA-seq data(GEO: GSE137869) were re-analyzed here. **i** Box plots of log-transformed gene expression of *pclaf* in clusters of BMMs from young and old rats. **j** Representative images of co-localization of PCLAF (green) and F4/80 (red) staining in distal femurs of young, old and exercise old mice (scale bars, 50 μm, *n* = 4). **k**–**n** Correlation analysis of expression levels of *PCLAF* and *IL8* (**k**), *IL6* (**l**), *MCP1* (**m**), *CXCL2*(**n**) in human bone marrow from GTEx dataset (*n* = 70). **o**, **p** Correlation analysis of levels of PCLAF and IL8 (**o**), IL6 (**p**) in human bone marrow supernatant. Data are shown as the mean ± SD. *P < 0.05, **P < 0.01, ***P < 0.001 by two-way ANOVA (**e**, **f**), Wilcoxon test (**g**–**i**) or Pearson’s correlation analysis (**k**–**p**)

To figure out the critical regulators involved in bone aging, we analyzed our recent liquid chromatography-tandem mass spectrometry (LC-MS/MS) data of young (3 months) and old (24 months) bone marrow supernatant and identified the top 40 upregulated factors (Fig. 2a). The majority among them exhibited lower expression in the bone or bone marrow according to a gene expression public database. Of the remaining candidates, PCLAF, which plays an essential role in cell proliferation, apoptosis and cell cycle regulation, gained our attention. PCLAF was found to participate in cell proliferation and regulate inflammatory pathways in diseases,^31,32^ but little is known about bone metabolism. We then examined PCLAF expression in a variety of organs and found it was predominantly expressed in the bone tissue and bone marrow (Fig. 2b, c). Next, we confirmed PCLAF increased with aging while decreased after exercise, whose changing trends were consistent with the senescence of BMAds (Fig. 2d–f and Fig. S1a, b).

To verify the cellular origin of PCLAF, we screened for the expression of *Pclaf* through previous scRNA-seq analysis of bone marrow cells of ground vs running mice and young vs old rats.^5,24^ PCLAF was highly expressed in myeloid cells, including granulocyte-monocyte progenitor (GMP), neutrophil and bone marrow macrophages (BMMs) (Fig. 2g, h and Fig. S1c) and we also identified 5 transcriptionally distinct clusters in primary bone marrow macrophage populations, where cluster 4 and cluster 5 had enrichment of *Pclaf* in old mice compared to young mice (Fig. 2i and Fig. S1d). We further detected the mRNA and protein expression of PCLAF in a variety of cells and excluded its origin from other skeletal resident cells (Fig. S1e–h). Thus, we preliminarily hypothesized that PCLAF originated from BMMs and confirmed this through detecting the co-localization of PCLAF and F4/80 (a marker of macrophages) (Fig. 2j). In line with above data, PCLAF expression in the BMMs increased during aging while decreased after exercise in both mRNA and protein levels (Fig. S1i–k). Moreover, *Pclaf* was also regulated by *Peizo1*(a mechanosensitive ion channel protein) as indicated by increased expression of *Pclaf* when *Peizo1* was knockdown (Fig. S1l). PCLAF was previously found to be in centrosomes in the perinuclear region.^33^ However, here we found that PCLAF was distributed in the centrosomes as well as the cytoplasm (Fig. S1m). In addition, we also observed a high level of His-PCLAF concentration in a concentrated cell culture medium of HEK293T cells transfected with His-*Pclaf* plasmid (Fig. S1n). Moreover, PCLAF was detected in the concentrated culture medium of RAW264.7 (Fig. S1o). All these data strongly indicated PCLAF was a secretory factor.

Interestingly, PCLAF was accumulated during aging while decreased after exercise, which resembled the changing trends of SASPs. We further found that there were direct positive relationships between the expression levels of *PCLAF* and *IL8, IL6, MCP1*, and *CXCL2* in the bone marrow according to the data of Genotype-Tissue Expression project (GTEx) (Fig. 2k–n). Additionally, we collected bone marrow supernatant from clinical patients and found a positive correlation between PCLAF and IL8 and IL6 (Fig. 2o, p), suggesting PCLAF was likely to induce skeletal aging. Together, our data above exhibit that decreased BMMs-derived PCLAF may be involved in the benefits of exercise on skeletal aging.

### Macrophages-derived PCLAF induces BMAds senescence

**Fig. 3:**
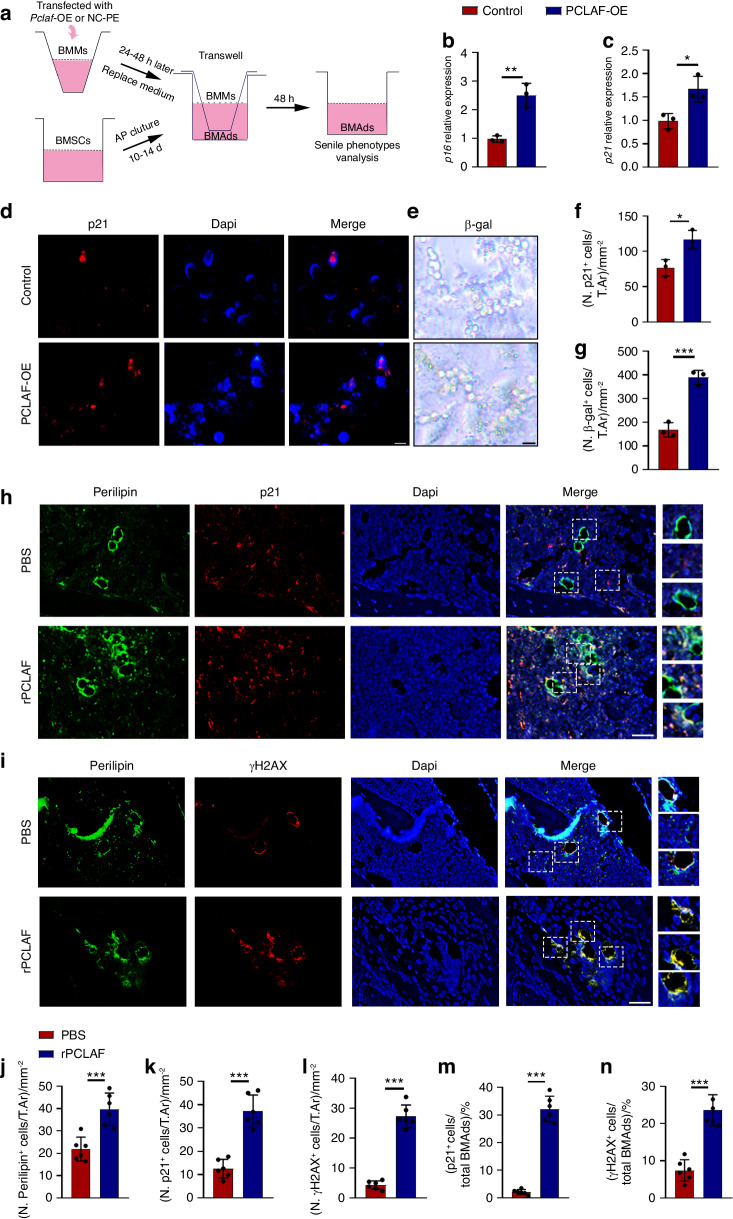
Macrophages-derived PCLAF induces BMAds senescence. **a** Pattern diagram of BMSCs-derived adipocytes co-cultured with BMMs transfected with plasmid-*Pclaf* or control plasmid. **b**, **c** QPCR analysis of *p16* (**b**) and *p21* (**c**) of BMSCs-derived adipocytes. **d** Representative images of immunofluorescence staining of p21 of BMSCs-derived adipocytes (scale bar, 50 μm; *n* = 3). **e** Representative images of immunofluorescence staining of β-gal staining (scale bar, 50 μm; *n* = 3). **f**, **g** Quantification of the percentage of p21^+^ cells (**f**) and β-gal^+^ staining cells (**g**). **h**, **i** Representative images of co-localization staining of p21 (**h**) and γH2AX (**i**) with perilipin (scale bar, 50 μm; *n* = 6). **j**–**n** Quantification of the number of perilipin^+^ cells per mm^2^ tissue area (N. Perilipin^+^ cells, **j**), the number of p21^+^ cells per mm^2^ tissue area (N. p21^+^ cells, **k**), the number of γH2AX ^+^ cells per mm^2^ tissue area (N. γH2AX ^+^ cells, **l**), the percentage of p21^+^ cells in total perilipin^+^ cell population ((p21^+^ cells/total BMAds)/%, **m**) and the percentage of γH2AX ^+^ cells in total perilipin^+^ cell population ((γH2AX ^+^ cells/total BMAds)/%, **n**) (*n* = 6). Data are shown as the mean ± SD. *P < 0.05, **P < 0.01, ***P < 0.001 by Student’s *t*-test (**b**, **c**, **f**, **g**, **j**–**n**)

Based on the above results, we examined the effects of BMM-derived PCLAF on bone marrow adipocytes. Firstly, we co-cultured BMSC-derived adipocytes with BMMs transfected with plasmid-*Pclaf* (*Pclaf*-OE) or control plasmid (control) (Fig. 3a). QPCR and ELISA quantification confirmed successful overexpression of *Pclaf* in BMMs (Fig. S2a, b). As expected, BMSC-derived adipocytes co-cultured with *Pclaf*-OE-BMMs exhibited senescent phenotype as indicated by higher expression of *p16, p21* and more p21^+^ and SA β-gal^+^ adipocytes compared to the control group (Fig. 3b–g). To investigate whether soluble PCLAF could directly induce BMAds senescence, we utilized human recombinant PCLAF (rPCLAF) to treat BMSC-derived adipocytes and found consistent results (Fig. S2c–f).

To further test the effects of PCLAF in vivo, we intramedullary injected rPCLAF with a dose of 0.2 mg/kg or PBS once per week for four weeks in 3-month-old mice (Fig. S2g). Corresponding to the in-vitro experiments, rPCLAF held the same ability to induce BMAds senescence in vivo as evidenced by higher expression of γH2AX (a DNA damage marker) and p21 in BMAds (Fig. 3h–n). Collectively, our results suggest that soluble PCLAF enables to induce BMAds senescence.

### PCLAF-mediated BMAds senescence induces bone loss

**Fig. 4:**
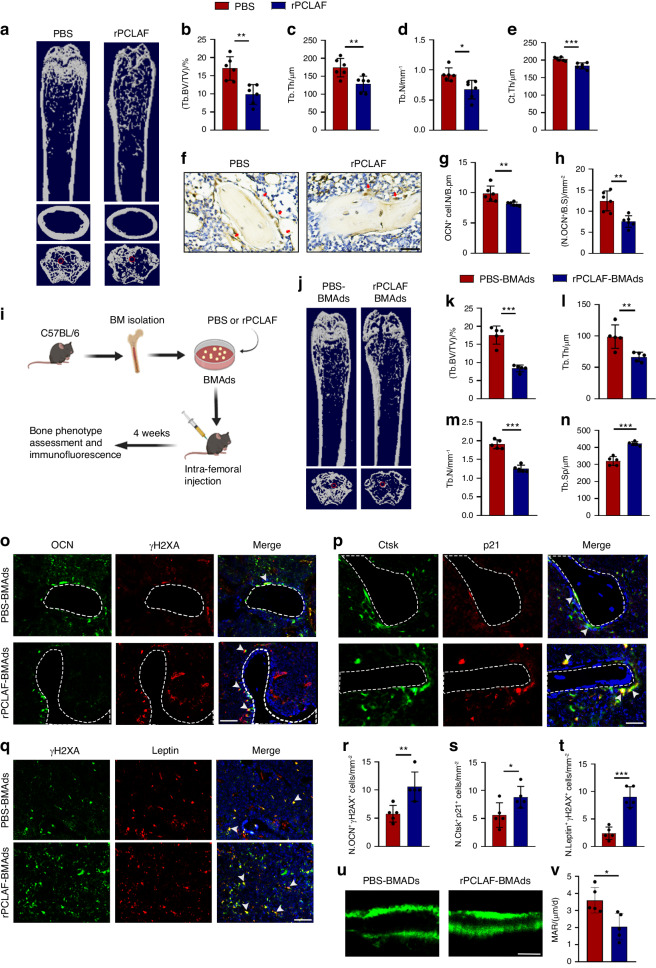
PCLAF-mediated BMAds senescence induces bone loss. **a** Representative image of Micro-CT of mice injected with rPCLAF or PBS (*n* = 6). **b**–**e** Quantitative analysis of Tb.BV/TV (**b**), Tb.Th (**c**), Tb.N (**d**), Ct.Th (**e**) (*n* = 6). **f**–**h** Representative images of osteocalcin (OCN) staining (**f**) and quantification (**g**, **h**) of the number of OCN^+^ cells (red arrows) in femurs (scale bar, 50 μm; *n* = 6). **i** Pattern diagram of experimental design for BMAds transplantation. **j** Representative images of Micro-CT of mice transplanted with PBS-treated or rPCLAF treated BMAds (*n* = 5). **k**–**n** Quantitative analysis of BV/TV (**k**), Tb.Th (**l**), Tb.N (**m**), Tb.Sp (**n**) (*n* = 5). **o**–**q** Representative images of co-localization staining of OCN (**o**), Ctsk (**p**) and leptin (**q**) with senescent markers (p21, γH2AX) (scale bar, 50 μm; *n* = 5). **r**–**t** Quantification of numbers of double-positive cells per mm^2^ tissue area, shown as N. OCN^+^ γH2AX^+^ cells (**r**), N. Ctsk^+^ p21^+^ cells (**s**) and N. Leptin^+^ γH2AX^+^ cells (**t**), respectively (*n* = 5). **u**, **v** Representative images of calcein double-labeling (**u**) and analysis of mineral apposition rates (MARs) (**v**) of trabecular bone in femurs (scale bars, 50 mm, *n* = 5). Data are shown as the mean ± SD. *P < 0.05, **P < 0.01, ***P < 0.001 by Student’s *t*-test (**b**–**e**, **g**, **h**, **k**–**n**, **r**–**t**, **v**)

To figure out the direct effect of PCLAF on skeletal aging, we conducted Micro-CT and found rPCLAF-injected mice showed a decreased bone mass when compared to the vehicle-treated group (Fig. 4a–e), along with reduced colony-formation ability of BMSCs (Fig. S3a, b). Furthermore, rPCLAF-injected mice also had lower osteoblastic and osteoclastic functions (Fig. 4f–h and Fig. S3c–e).

We then wondered whether PCLAF-mediated BMAds senescence was the key regulator of skeletal aging. Thus, we isolated adipocytes from 3-month-old mice and followed by the treatment of rPCLAF or PBS for three days. Then, rPCLAF-treated conditioned medium (rPCLAF-CM) and PBS-treated conditioned medium (PBS-CM) were collected and applied to culture BMSCs and BMMs (Fig. S3f). We detected higher levels of SASP factors in rPCLAF-CM compared to PBS-CM (Fig. S3g). Additionally, we observed more β-gal^+^ BMSCs and higher expression of *p16* and *p21* in the rPCLAF-CM compared to the PBS-CM treated group (Fig. S3h–k). Moreover, BMSCs-differentiated osteoblasts treated with rPCLAF-CM exhibited higher expression of *p16* and *p21* and impaired osteogenic differentiation, representing lower mineralization and expression of osteogenic genes (Fig. S3l–p). In contrast, adipogenic differentiation was enhanced with the treatment of rPCLAF-CM than PBS-CM as indicated by more lipid droplets and higher expression of adipogenic genes (Fig. S3q–s). Furthermore, the TRAP^+^ cells and expression of osteoclastogenic genes were decreased in osteoclasts with the treatment of rPCLAF-CM than PBS-CM (Fig. S3t–v). Together, our data suggest that PCLAF-mediated BMAds senescence disrupts the function of bone resident cells.

To test this in vivo, we performed BMAds transplantation experiments. We firstly collected BMAds from 3-month-old mice and confirmed there was an enriched population of BMAds rather than osteogenic or immune cells (Fig. S3w). To identify how long the transplanted adipocytes would persist in recipient mice, we labeled BMAds with adenovirus (HBAD-EGFP) and then transplanted them into the femoral bone marrow cavity of recipient mice. After 1 month, EGFP^+^ BMAds were also detected in recipient mice (Fig. S3x, y), suggesting the stable persistence of BMAds within one month. Next, we treated the isolated BMAds with rPCLAF or PBS for 3 days. Then, rPCLAF-treated and PBS-treated BMAds were transplanted into recipient mice (Fig. 4i). After 4 weeks, we observed mice transplanted with BMAds treated with rPCLAF (rPCLAF-BMAd) had a significant bone loss compared with those transplanted with the same number of BMAds treated with PBS (PBS-BMAd) (Fig. 4j–n). More γH2AX^+^ osteoblastic/ BMSCs and p21^+^ osteoclastic cells were observed in rPCLAF-BMAd mice than in PBS-BMAd mice (Fig. 4o–t). Finally, calcein double labeling revealed that rPCLAF-BMAd recipient mice showed lower trabecular mineral apposition rates (MARs) compared with mice transplanted with PBS-treated BMAds (Fig. 4u, v). In general, our data suggest PCLAF-mediated BMAds senescence decreases bone mass.

### Macrophages-derived PCLAF, an adipocyte senescence inducer, impaired bone homeostasis

To further verify the role of PCLAF in vivo, we generated a conditional-knockout mouse model with deletion of the *Pclaf* gene in macrophages (*Pclaf*-Lyz2-CKO) using *Pclaf*-floxed mice crossed with Lyz2-Cre mice (Fig. S4a, b). An apparent reduction of *Pclaf* both in the bone marrow supernatants and BMMs confirmed the successful deletion of *Pclaf* in macrophages (Fig. S4c–e). Under homeostatic conditions, no significant differences in body weight were observed between 9-, 15-month-old *Pclaf*-Lyz2-CKO mice and control littermates, which excluded the effect of body weight on the skeleton (Fig. S4f, g).

**Fig. 5:**
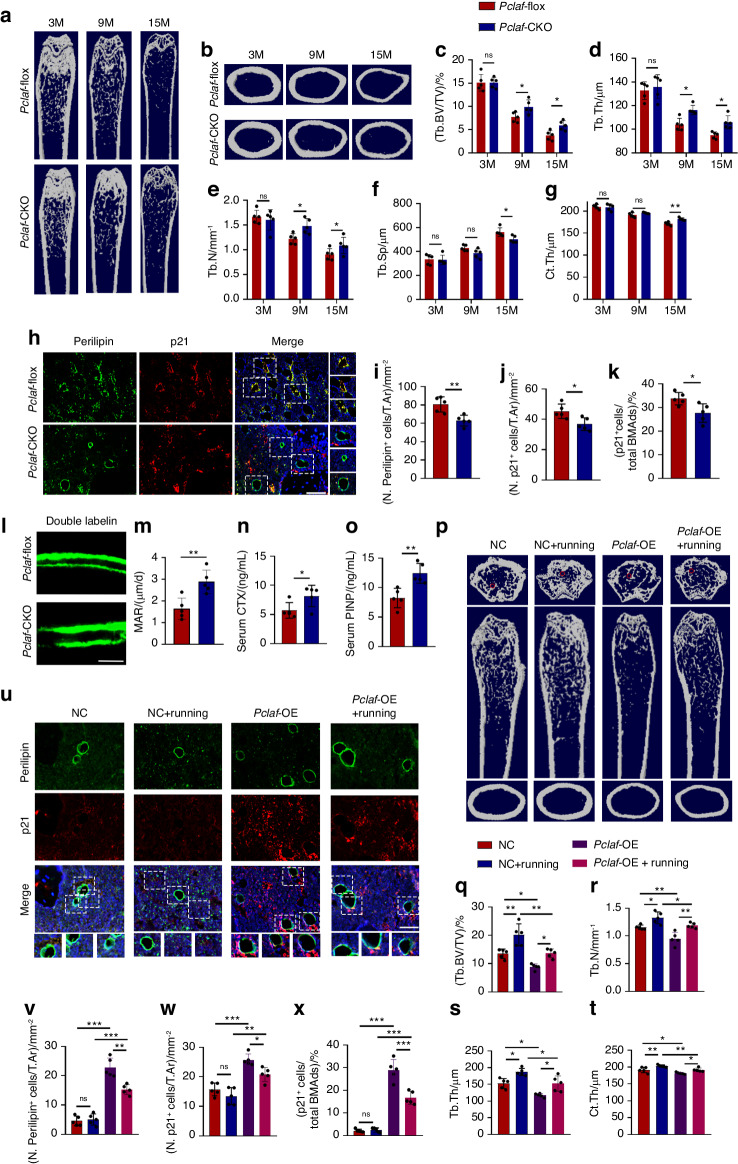
Macrophages-derived PCLAF, an adipocyte senescence inducer, impaired bone homeostasis. **a**, **b** Representative images of Micro-CT of 3-month, 9-month and 15-month-old *Pclaf*^flox/flox^ or *Pclaf*-Lyz2-CKO mice (*n* = 5). **c**–**g** Quantitative analysis of Tb.BV/TV (**c**), Tb.Th (**d**), Tb.N (**e**), Tb.Sp (**f**), Ct.Th (**g**) (*n* = 5). **h** Representative images of co-localization staining of p21 and perilipin (scale bar, 50 μm; *n* = 5). **i**–**k** Quantification of the number of perilipin^+^ cells per mm^2^ tissue area (N. Perilipin^+^ cells, **i**), the number of p21^+^ cells per mm^2^ tissue area (N. p21^+^ cells, **j**), and the percentage of p21^+^ cells in total perilipin^+^ cell population ((p21^+^ cells/total BMAds)/%, **k**) (*n* = 5). **l**, **m** Representative images of calcein double-labeling (**l**) and analysis of MARs (**m**) of trabecular bone in femurs (scale bars, 50 mm, *n* = 5). **n** Serum CTX level of 15-month-old *Pclaf*^flox^ and *Pclaf*-Lyz2-CKO mice (*n* = 5). **o** Serum PINP level of 15-month-old *Pclaf*^flox^ and *Pclaf*-Lyz2-CKO mice (*n* = 5). **p**–**t** Representative images (**p**) of Micro-CT and quantitative analysis of Tb.BV/TV (**q**), Tb.N (**r**), Tb. Th (**s**), Ct.Th (**t**) of mice intra-femoral injected with AAV-F4/80-*Pclaf* (*Pclaf*-OE) or AAV-F4/80-empty (NC), followed by exercise intervention or not (*n* = 5). **u** Representative images of co-localization staining of p21 and perilipin (scale bar, 50 μm; *n* = 5). **v**–**x** Quantification of the number of perilipin^+^ cells per mm^2^ tissue area (N. Perilipin^+^ cells, **v**), the number of p21^+^ cells per mm^2^ tissue area (N. p21^+^ cells, **w**), and the percentage of p21^+^ cells in total perilipin^+^ cell population ((p21^+^ cells/total BMAds), **x**) (*n* = 5). Data are shown as the mean ± SD. *P < 0.05, **P < 0.01, ***P < 0.001 by Student’s *t*-test (**i**–**k**, **m**–**o**) or two-way ANOVA (**c**–**g**, **q**–**t**, **v**–**x**)

Though young mice (3 months) had similar bone mass, *Pclaf*-Lyz2-CKO mice showed delayed age-related bone loss compared to age-matched *Pclaf*^flox/flox^ controls in 9- and 15-month-old mice (Fig. 5a–g and Fig. S4h). Additionally, with the increase of age, adipocytes in bone marrow also show a gradual aging state (Fig. S4i). However, 15-month *Pclaf*-Lyz2-CKO mice exhibited fewer p16^+^/p21^+^ adipocytes and increased colony-formation ability of BMSCs compared to *Pclaf*^flox/flox^ controls (Fig. 5h–k and Fig. S4j–m). Improved bone remodeling in *Pclaf*-Lyz2-CKO mice was also confirmed by elevated osteoblasts, osteoclasts, MARs, serum CTX and procollagen type I N-propeptide (PINP) levels compared to control *Pclaf*^flox/flox^ mice (Fig. 5l–o and Fig. S4n–r). In general, these data suggest that depletion of *Pclaf* in the BMMs inhibits BMAds senescence and delays bone aging.

To further investigate the link between PCLAF and exercise-alleviated bone aging, we utilized F4/80 promoter-driven adeno-associated virus (AAV) to establish a *Pclaf* overexpression model in vivo, which mimicked PCLAF accumulation in BMMs during aging. AAV-F4/80-*Pclaf* (*Pclaf*-OE) or AAV-F4/80-empty (used as control) was injected into the bilateral bone marrow of 3-month-old mice. 4 weeks later, these mice were randomly divided into four groups with AAV-F4/80-*Pclaf* or AAV-F4/80-empty either undergoing running intervention or not (Fig. S5a). QPCR and ELISA analysis confirmed successful overexpression of *Pclaf* (Fig. S5b, c). In addition, running partially decreased the level of PCLAF compared to the overexpression group (Fig. S5c).

As expected, mice with PCLAF overexpression displayed decreased bone mass compared to the control group, while the bone loss was restored after running (Fig. 5p–t). Consistently, we observed a decline in the colony-formation ability of BMSCs, the numbers of osteoblast and osteoclast cells in *Pclaf*-overexpression mice compared to *Pclaf*^flox/flox^ mice (Fig. S5d–j). Of note, exercise partially abolished these alterations in *Pclaf*-overexpression mice (Fig. S5d–j). Consistently, we observed that exercise alleviates BMAds senescence induced by *Pclaf* overexpression, as evidenced by fewer p21^+^ BMAds (Fig. 5u–x). In general, our data suggest exercise inhibits PCLAF secretion and thereby delays BMAds senescence and bone aging.

### PCLAF binds to the ADGRL2 receptor of BMAds to accelerate skeletal aging

**Fig. 6:**
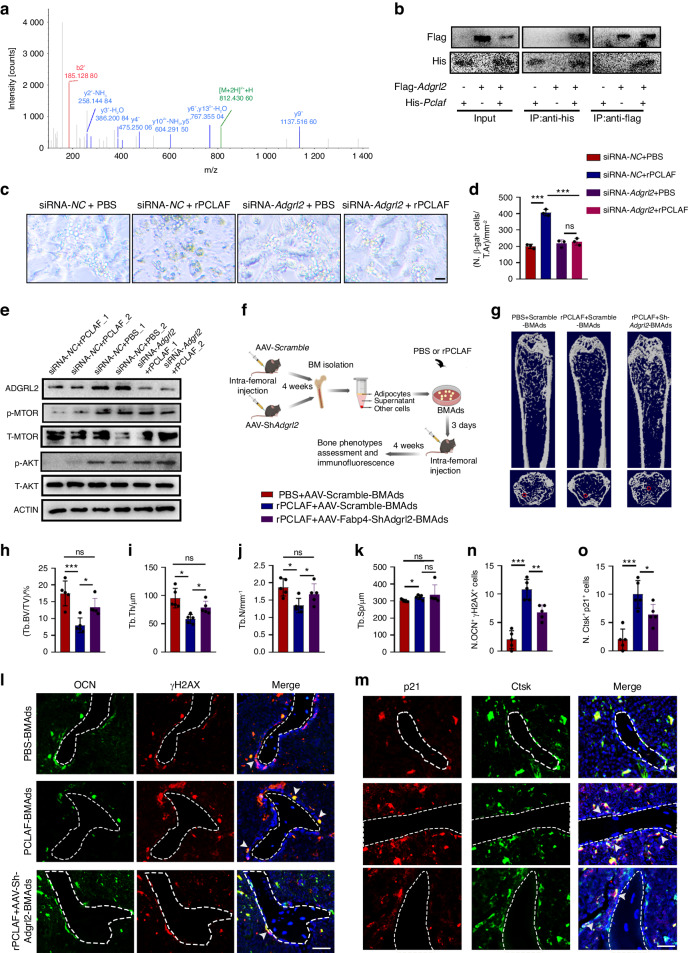
PCLAF binds to the ADGRL2 receptor of BMAds to accelerate skeletal aging. **a** LC-MS/MS analysis of identified ADGRL2. **b** Co-immunoprecipitation (IP) analysis of binding between His-PCLAF and Flag-ADGRL2. **c**, **d** Representative images (**c**) and quantification of SA β-gal (**d**) staining of BMSCs-derived adipocytes transfected with siRNA-*Adgrl2* or siRNA-*NC* in the presence or absence of rPCLAF (*n* = 3). **e** Representative western blot of AKT, mTOR protein/phosphorylation level of BMSCs-derived adipocytes transfected with siRNA-*Adgrl2* or siRNA-NC in the presence or absence of rPCLAF. **f** Pattern diagram of experimental design for transplantation of BMAds from AAV-Fabp4-Scramble or AAV-Fabp4-Sh*Adgrl2* mice and then treated with rPCLAF or PBS. **g** Representative images of Micro-CT of mice in **f** (*n* = 5). **h**–**k** Quantitative analysis of Tb.BV/TV (**h**), Tb.Th (**i**), Tb.N (**j**), Tb,Sp (**k**) (*n* = 5). **l**, **m** Representative images of co-localization staining of OCN (**l**) and Ctsk (**m**) with senescent markers (p21 and γH2AX) (*n* = 5). **n**, **o** Quantification of double positive cells per mm^2^ tissue area, shown as N. Ocn^+^γH2AX^+^ cells (**n**) and N. Ctsk^+^p21^+^ cells (**o**) (scale bar, 50 μm; *n* = 5). Data are shown as the mean ± SD. *P < 0.05, **P < 0.01, ***P < 0.001 by two-way ANOVA (**d,** **h**–**k**, **n**, **o**)

To identify potential receptors of PCLAF in BMAds, we conducted mass spectrometry with cell lysates of BMAds that were incubated with His-labeled PCLAF (Fig. S6a). Among six high-scoring candidates, adhesion Class G-protein-coupled receptor (ADGRL2, formerly termed latrophilin 2) exhibited the highest expression (Fig. 6a and Fig. S6b). ADGRL2 is an orphan receptor that belongs to the adhesion G-protein-coupled receptors (GPCRs) which was reported to be involved in adipose tissue metabolism.^34^

To identify whether PCLAF bound to ADGRL2, we transfected FLAG-ADGRL2 and His-PCLAF plasmids into HEK293T cells and then collected the cell lysates for immunoprecipitation (IP) by an anti-FLAG antibody, which was followed by western blotting using an His antibody. We observed a strong band of FLAG staining in His immunoprecipitants (Fig. 6b). We also conducted an IP assay using an anti-His antibody in the cell lysate followed by western blotting analysis using an anti-FLAG antibody. The results also supported the interaction between PCLAF and ADGRL2 (Fig. 6b).

To demonstrate that ADGRL2 mediated the effects of PCLAF on BMAds, we transfected bone marrow adipocytes with *Adgrl2* siRNA or scramble siRNA with or without treatment of rPCLAF. QPCR for *Adgrl2* proved successful knockdown of *Adgrl2* (Fig. S6c). Our findings revealed that treatment with rPCLAF resulted in the adipocytes senescence as indicated by a greater number of SA β-gal^+^ cells, while this failed to occur with deficiency of *Adgrl2* (Fig. 6c, d). These findings suggest that ADGRL2 functions as a receptor for PCLAF and mediates the effects of PCLAF on promoting adipocytes senescence.

To further elucidate the mechanism of how PCLAF affected bone metabolism, we performed RNA-seq analysis of BMAds treated with rPCLAF or PBS. Among the top 20 enrichment pathways, the AKT signaling pathway exhibited a remarkable impact (Fig. S6d). Further studies verified that rPCLAF inhibited this pathway as evidenced by decreased phospho-AKT substrate and phosphor-mTOR expression in a time- and dose-dependent manner (Fig. S6e, f). However, the effect of rPCLAF on phosphorylation of mTOR and AKT was blocked in SiRNA-*Adgrl2*-transfected BMAds (Fig. 6e). Together, we speculated that PCLAF bound to ADGRL2 and inhibited AKT-mTOR signaling to induce bone aging.

To demonstrate the effect of ADGRL2 in vivo, we intra-femoral injected AAV-Fabp4-Scramble or AAV-Fabp4-Sh*Adgrl2* into 3-month-old mice to specifically deplete *Adgrl2* in BMAds. After four weeks, rPCLAF and PBS were then injected into the bone marrow cavity per week for four weeks to form four groups, including PBS-treated AAV-Fabp4-Scramble mice (PBS-Scramble), rPCLAF-treated AAV-Fabp4-Scramble mice (rPCLAF-Scramble), PBS-treated AAV-Fabp4-Sh*Adgrl2* mice (PBS-AAV-Sh*Adgrl2*) and rPCLAF-treated AAV-Fabp4-Sh*Adgrl2* (rPCLAF-AAV-Sh*Adgrl2*) mice. rPCLAF-Scramble mice showed lower bone mass compared to PBS-Scramble mice while this effect was failed to occur in mice with BMAds-specific knockout of *Adgrl2* (Fig. S6g–k).

To further validate the direct effects of PCLAF on BMAds senescence and BMAds senescence-mediated secondary senescence in the bone marrow, we then isolated adipocytes from PBS-Scramble and AAV-Sh*Adgrl2* mice followed by the treatment of rPCLAF. Afterward, they were transplanted into 3-month-old mice (recipient) (Fig. 6f). Consistently, recipient mice transplanted with BMAds from control mice followed by the treatment of rPCLAF (rPCLAF-Scramble-BMAd) showed a reduced bone mass compared with those transplanted with the same number of BMAds from PBS-Scramble donors (PBS-Scramble-BMAd), which did not occur in mice transplanted with BMAds from AAV-Sh*Adgrl2* (rPCLAF+AAV-Sh*Adgrl2*-BMAd) mice (Fig. 6g–k). We also observed more senescent osteoblasts, osteoclasts and BMSCs in rPCLAF-Scramble-BMAd mice compared to PBS-Scramble-BMAd mice, while these were offset in rPCLAFAAV-Sh*Adgrl2*-BMAd mice (Fig. 6l–o and Fig. S6l, m). In general, our results support that ADGRL2 mediates the effect of PCLAF on bone homeostasis.

### PCLAF-neutralizing antibody improves skeletal health in old mice

Our above findings prompted us to investigate the impact of a neutralizing antibody targeting PCLAF. To this end, we generated six purified anti-PCLAF monoclonal antibodies and applied them to treat 3T3-L1 cells in the presence or absence of rPCLAF. Among all the candidates, PCLAF-NAb1 (hereafter referred to as PCLAF-NAb) exhibited significant effects on suppressing the expression levels of *p16* and *p21* and thus was chosen for further studies (Fig. S7a, b). Of note, PCLAF-NAb treatment blocked the AKT/mTOR pathway in BMSC-derived adipocytes (Fig. S7c).

**Fig. 7:**
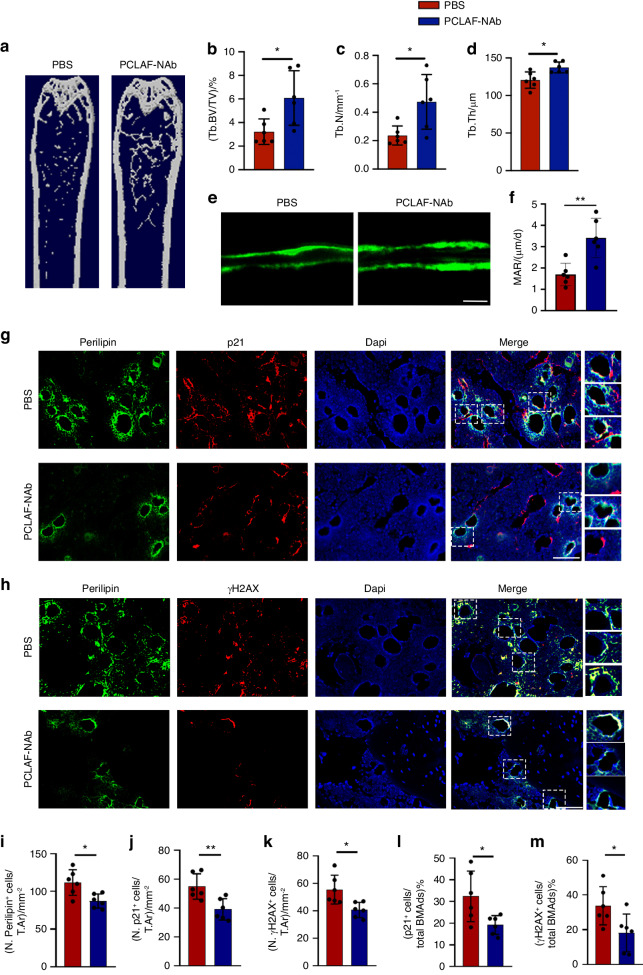
PCLAF-neutralizing antibody improves skeletal health in old mice. **a** Representative image of Micro-CT of 18-month-old mice treated with PBS or rPCLAF neutralizing antibody (PCLAF-NAb) (*n* = 6). **b**–**d** Quantitative analysis Tb.BV/TV (**b**), Tb.N(**c**), Tb.Th (**d**) (*n* = 6). **e**, **f** Representative images of calcein double-labeling (**e**) and analysis of MARs (**f**) of trabecular bone in femurs (scale bars, 50 mm, *n* = 6). **g** Representative images of co-localization staining of p21and perilipin (scale bar, 50 μm; *n* = 6). **h** Representative images of co-localization staining of γH2AX and perilipin (scale bar, 50 μm; *n* = 6). **i**–**m** Quantification of the number of perilipin^+^ cells per mm^2^ tissue area (N. Perilipin^+^ cells, **i**), the number of p21^+^ cells per mm^2^ tissue area (N. p21^+^ cells, **j**), the number of γH2AX^+^ cells per mm^2^ tissue area (N. γH2AX ^+^ cells, **k**), the percentage of p21^+^ cells in total perilipin^+^ cell population ((p21^+^ cells/total BMAds)/%, **l**) and the percentage of γH2AX^+^ cells in total perilipin^+^ cell population ((γH2AX ^+^ cells/total BMAds)/%, **m**) (*n* = 6). Data are shown as the mean ± SD. *P < 0.05, **P < 0.01, ***P < 0.001 by Student’s *t*- test (**b**–**d**, **f**, **i**–**m**)

Next, we injected PCLAF-Nab to 3-, and 18-month-old mice (C57BL/6 mice) for two months and proved successful neutralization of PCLAF in the bone marrow supernatant of old mice (Fig. S7d). PCLAF-NAb treatment increased bone mass in 18-month-old mice but not in 3-month-old mice, suggesting the therapeutic effect of PCLAF-NAb was account for the age-dependent accumulation of PCLAF (Fig. 7a–d, Fig. S7e–i). Moreover, treatment of PCLAF-NAb improved bone remodeling in old mice, as indicated by elevated MARs and quantification of osteoblasts and osteoclasts, when compared to PBS-treated old mice (Fig. 7e–f and Fig. S7j–p). Consistently, the administration of PCLAF-NAb resulted in fewer senescent BMAds(p21^+^, p16^+^) in bone marrow (Fig. 7g–m and Fig. S7q–r). Together, our data reveal that PCLAF-Nab is a potential way for the treatment of age-related osteoporosis.

## Discussion

Bone marrow is composed of immune cells, skeletal lineage cells, and bone marrow adipocytes.^35^ The senescent immune cells and skeletal lineage cells were reported to secrete abundant proinflammatory cytokines, and chemokines during aging, which impaired bone homeostasis.^36–38^ However, the roles of senescent BMAds were elusive even though BMAds account for a nonnegligible part of bone marrow.^39^ Consistent with peripheral adipose senescence as an early onset event during aging and spread the senescence signaling to neighbor or remote cells and tissues,^40,41^ recent studies have identified senescent BMAds as the initiator of bone loss under the actions of glucocorticoids.^16^ Here, we investigated that aging induced BMAds senescence impaired bone remodeling. Thus, differing from the well-established roles of BMAds, such as adipogenic differentiation or lipotoxicity,^42–44^ we expanded the roles of BMAds senescence during aging process and exercise intervention.

Importantly, targeting adipose tissue outside the bone marrow is applied to treat metabolic disorders, such as cardiovascular disease and fatty liver.^45,46^ It is intriguing whether targeting BMAT can exert benefits on homeostasis of the skeletal niche. Previous studies showed that regulation of bone fat through affecting immune cells in the bone marrow showed benefits in bone homeostasis.^47,48^ For example, our previous study found that grancalcin neutralizing antibody targeting senescent BMMs decreased marrow fat accumulation while lipolytic factor reticulocalbin-2 shrunk the BMAds, thus helping to alleviate age-related osteoporosis.^5,24^ Of note, our data confirmed the balance between bone resorption and bone formation from the new perspective of BMAds senescence.

Exercise mediated its benefits to bone health mainly through its function of promoting osteoblast-osteoclast crosstalk and secreting anti-inflammatory factors.^49,50^ Our previous study found exercise promoted the secretion of reticulocalbin-2, thus accelerating lipolysis and shrinking the adipocytes in the bone marrow.^24^ However, how BMAds regulate bone restructuring remains unclear. In our study, we identified a BMM-derived factor PCLAF, which increased with aging but was abolished by exersise. Furthermore, we found that exercise promoted bone turnover during aging by slowing down PCLAF-induced senescence of BMAds, which offered a new insight into the mechanism of physical exercise benefiting skeletal health. BMAds act as a special cell type compared to peripheral adipocytes.^51–53^ They may secrete senescent factors that mediate bone aging and lead to reduced osteogenesis and increased osteoclasts.^54,55^ Moreover, some adipocyte-secreted adipokines reduce osteogenesis and osteoclasts.^54,56^ Our future studies will focus on the detailed way of BMAds affecting bone resorption or formation.

To date, the physiological role of PCLAF is unclear. It is a 15 kD protein encoded by the KIAA0101 gene and it can interact with PCNA, which is a necessary molecule of DNA replication and DNA repair.^57^ Previous studies showed that PCLAF was mainly expressed in the nucleus.^32^ However, we verified its secreted characteristic through its presence in serum, bone marrow supernatant as well as cell culture medium. Mechanistically, PCLAF accelerated the senescence of BMAds via binding to the ADGRL2 receptor and inhibiting the AKT-mTOR signaling pathway. Of note, the activity of this signaling generally played a central role in regulating a variety of cellular processes ranging from cell survival to aging.^58,59^ Here, we found that PCLAF-mediated inhibition of AKT signaling pathway increased the expression of senescent markers. Moreover, previous studies reported ADGRL2 receptor is mainly related to tumorigenesis.^60^ In our study, the newly identified ADGRL2 receptor mediated the process of adipocyte senescence, which might offer the choice for the pharmacotherapy development of receptor molecules for the improvement of the decline of bone mass.

The skeletal homeostasis is maintained by a dynamic equilibrium of bone formation and bone resorption. The canonical osteoporosis drugs take effect mainly by promoting bone formation or inhibiting bone resorption.^61^ However, parathyroid hormone (PTH), a clinically utilized effective drug for osteoporosis,^62^ exhibited unique characteristics that promote both bone formation and bone resorption as well as regulating BMATs,^13,63–65^ which resembles the function of PCLAF-Nab in our study. These findings reminded us to think about the decreased bone remodeling during aging. On the contrary to the effects of PTH, aging affects both bone formation and resorption whose overall effect is an anti-osteoanabolic effect due to the weaker effects on formation.^66–68^ Thus, we validated the potential therapeutic effects of PCLAF-neutralizing antibody in age-related bone loss, which offered a new strategy for the exploration of anti-osteoporosis drugs.

Together, we revealed the mechanism by which exercise delayed aging by removing PCLAF to alleviate BMAds-mediated bone aging. We elucidated PCLAF as a negative regulator of BMAds and bone remodeling. More importantly, we verified that the PCLAF-neutralizing antibody exhibited beneficial effects on bone health in old mice, offering more opportunities for treating age-related osteoporosis.

## Materials and methods

### Animal models

C57BL/6 J mice were procured from Hunan SJA Laboratory Animal Company (Hunan, China). *Pclaf*-floxed mice were generated by inserting two flox sequences at the terminals of *Pclaf* exon 3. For genotyping, genomic DNA was extracted from tail tips, and the primers of genotyping for *Pclaf*-floxed mice were: *Pclaf*-L-loxp-F: CACATGATTCTGGGTTCAATCTCT; *Pclaf*-L-loxp-R: GACCTTATTCGTGCCACAACACAT; *Pclaf*-R-loxp-F2: TTGCAATCCTCTGCCTCGACT; *Pclaf*-R-loxp-R2: GTGGATTCGGACCAGTCTGA. *Lyz2*-Cre mice were purchased from the cyagen company. The primers for Lyz2-Cre mice were: Lyz2-cre-F1:CTTGGGCTGCCAGA ATTTCTC; Lyz2-cre-R1: CCCAGAAATGCCAGATTACG; Lyz2-cre-F2:CTTGGGC TGCCAGAATTTCTC; Lyz2-cre-R2:TTACAGTCGGCCAGGCTGAC. The myeloid-cell-specific *Pclaf* knockout mice were generated by interbreeding *Pclaf*-floxed mice and *Lyz2*-Cre mice. *Pclaf*-floxed littermates were utilized as controls.

All mice were maintained in a standard, specific pathogen-free facility at the Laboratory Animal Research Center of Central South University, with a controlled temperature (22–24 °C), a 12 h dark/light cycle (07:00 to 19:00 light on), and ad libitum access to standard food (Hunan SJA Laboratory Animal Company, China) and water. Environmental enrichments were provided to ensure their well-being. Mice were used for in-house mating to generate the required number of animals for experiments as indicated in the figure legends. Only male mice were used. All animal care protocols and experiments were reviewed and approved by the Animal Care and Use Committees of the Laboratory Animal Research Center at Xiangya Medical School of Central South University.

### BMSCs isolation

Bone marrow cells were flushed from tibias and femurs with a 1 mL syringe by using ice-cold α-MEM, then dispelled into single cell and seeded in culture 10 cm dish. The adherent cells were then incubated with phycoerythrin (PE)-, FITC-, peridinin chlorophyll protein (PerCP)- and allophycocyanin (APC)-conjugated antibodies that recognized mouse Sca-1 (BioLegend, 108108, 1:100), CD29 (BioLegend, 102206, 1:100), CD45 (BioLegend, 103132, 1:100), and CD11b (BioLegend, 101226, 1:100) for 20 min at 4 °C. The Sca-1^+^ CD29^+^ CD45^-^ CD11b^-^ cells were sorted as BMSCs by FACS (BD Biosciences), and were used for the further study.

### Bone marrow monocytes and macrophages isolation

Bone marrow cells were extracted from male mice tibias and femurs and cultured in α-MEM with 10% fetal bovine serum, 100 U/mL penicillin, 100 μg/mL streptomycin overnight. The floating cells were then collected to obtain monocytes and macrophages through adding 50 ng/mL M-CSF (R&D Systems) in the culture medium. To obtain preosteoclasts and mature osteoclasts, the monocytes and macrophages were then incubated with 30 ng/mL M-CSF and 60 ng/mL RANKL (462-TEC-010, Novus Biologicals) for 8 days. Following this, Alizarin Red staining were performed as described previously.^4^ The cells were fixed with 4% paraformaldehyde and TRAP (G1492-4, solarbio) staining was performed to detect osteoclastic differentiation according to the manufacturer’s instructions.

### Osteogenic differentiation assay

To induce osteoblastic differentiation, BMSCs were cultured in 6/12-well plates with culture medium consisting of α-MEM supplemented with 10% fetal bovine serum, 0.1 mmol/L dexamethasone, 10 mmol/L beta-glycerol phosphate, and 50 mmol/L ascorbate-2-phosphate, and cells were incubated for 21 days. Following this, Alizarin Red staining were performed as described previously.^44^ In brief, cells were fixed with 4% paraformaldehyde and stained them with 2% Alizarin red solution (Sigma-Aldrich). After images capture, 10% Hexadecylpyridinium chloride solution was added to the culture dish, and the supernatant was collected and measured OD value at 405 nm.

### Adipogenic differentiation assay

To induce adipogenic differentiation of BMSCs in vitro, BMSCs treated with α-MEM containing 10% fetal bovine serum, 0.5 mmol/L 3-isobutyl-1-methylxanthine, 5 μg/mL insulin, and 1 μmol/L dexamethasone and cells were incubated for 10 days. Following this, cells were fixed with 4% paraformaldehyde and Oil Red O (Sigma-Aldrich) staining was performed to detect lipid in mature adipocytes.

### Transwell experiments

BMSCs were isolated and induced for adipogenic differentiation for 10–14 days as described above. Then they were seeded at density of 10^5^ cells per well in 12-well plate in α-MEM with 10% fetal bovine serum, 100 U/mL penicillin, 100 μg/mL streptomycin. BMMs were acquired as described above. And they were transfected with *Pclaf*-OE and control plasmid for 24–48 h. Next, Transwell (Corning, 3401) permeable polycarbonate membrane 0.4 μm pore size Transwell inserts were added to wells intended for co-culture. Above BMSCs-differentiated BMAds were seeded to each Transwell inserts. After 48 h, BMAds were collected for further examinations.

### BMAds isolation

Mature BMAds were isolated directly from bone marrow of mice according to previously described protocols.^16,69^ Briefly, femurs and tibias were harvested from the mice, and the bone ends were trimmed. The bones were then placed in a small microcentrifuge tube (0.6 mL) that had been cut open at the bottom. This assembly was inserted into a larger microcentrifuge tube (1.5 mL). Fresh bone marrow was extracted by quick centrifugation. The collected bone marrow was treated with the RBC lysing buffer, followed by centrifugation (3 000 r/min, 5 min, RT). The adipocytes, forming a top layer, were carefully collected from the tube and washed three times with PBS.

### Cell transplantation

For the validation of adipocytes persistence, bone marrow adipocytes were isolated according to previously described protocols^16^ and labeled with HBAD-EGFP adenovirus. Afterwards, they were transplanted into femurs of 3-month-old mice. After 1 month, femurs were collected to detect the immunofluorescence intensity of EGFP to reflect the persistence of transplanted adipocytes.

For transplantation, bone marrow adipocytes were isolated from AAV-FABP4-Sh*Adgrl2*, AAV-Scramble mice and C57BL/6 mice. After being treated with rPCLAF or PBS for 3 days, they were transplanted into 3-month-old mice. Recipient mice were anesthetized, and a longitudinal incision was made on the front of the right knee to expose the patellar tendon. A 27 G needle was carefully inserted through the patellar tendon, positioned between the condyles of the femur, and gradually advanced with a twisting motion until reaching a depth of 2–4 mm within the bone. Confirmation of successful penetration was indicated by slow but consistent bleeding. Cell suspensions containing BMAds (3 × 10^3^) in 20 μL PBS were then slowly injected into the medullary space of the femur. The injection site was immediately sealed with bone wax, and the skin was sutured to complete the procedure.

### Colony formation assay

To perform the colony formation assay, cells were detached and centrifuged to obtain a cell pellet. The pellet was resuspended, enumerated, and adjusted to a concentration of 1 × 10^5^ cells/mL. This solution was then further diluted to a final concentration of 1 × 10^3^ cells/mL. An appropriate amount of the cell suspension was plated in a 6-well plate containing 4 mL of culture medium. The cells were uniformly dispersed and incubated in a 5% CO_2_ incubator for 2–3 weeks until colonies could be seen with the naked eye. Once visible, the cell culture was terminated, and the culture medium was discarded. To fix the colonies in place, the cells were treated with methanol for 15 min and then stained with crystal violet for an additional 10 min. The number of colonies visible to the naked eye was counted, and the colony rate was calculated as (colony number/number of seeded cells) x 100%.

### Recombinant PCLAF treatment

Human recombinant PCLAF (rPCLAF) was obtained from Sinobiological (Beijing, China, 10997-H07E). For animal studies, rPCLAF was administered intramedullary at a dose of 0.2 mg/kg/week bilaterally for one month. For cell experiments, rPCLAF was dissolved in PBS and applied at the indicated concentration and time.

### Intramedullary injection of adeno-associated virus

Recombinant adeno-associated serotype 8 viruses with F4/80 promoter for *Pclaf* overexpression in BMMs (AAV-F4/80-*Pclaf*) was purchased from Company (Shanghai, China). The AAV of Fabp4 promoter driven *Adgrl2* knockdown was generated by replacing the *Adgrl2* transcript sequence with shRNAs target for *Adgrl2*. An AAV-empty vector served as the control.

The experimental procedure involved depilation of hair near the knee joints of mice followed by making an incision on the matching skin around the joint. Subsequently, microscissors were employed to separate the muscle tissue and tweezers were used to displace the tendon towards the left side. Thereafter, a 29-gauge insulin syringe was carefully inserted into the bone marrow cavity from the distal femur, and 5 μL of 1.8 ×10^12^ μg/mL was infused into the bone marrow cavity. Subsequently, the muscle and tendon were repositioned to their original state, and the skin was sutured using a continuous stitch.

### ELISA

Bone marrow supernatants and bone marrow adipocytes supernatants were centrifuged at 12 000 r/min for 10 min to remove cellular debris. The ultrafiltration tubes are used for liquid concentration. Whole blood samples were centrifuged at 3 000 r/min for 10 min to get serum. For preparation of bone marrow supernatant, we exposed bone marrow of euthanized mice after cutting two ends of tibias and femurs and placed the samples for centrifugation for 15 min at 3 000 r/min and 4 °C to obtain bone marrow supernatants, which we then stored at −80 °C.

ELISA measurements were conducted using kits for PCLAF and CTX from CUSABIO (china), CCL2, TNF-α, IL-1b, IL-6, IL-8 form Multi Sciences LTD (Hangzhou, China), OPN, PINP from Abbexa (United Kingdom) according to the manufacturer’s instructions.

### Human bone marrow samples

Human bone marrow samples were obtained from 20 male patients with bone fracture, with ages ranging from 50 to 70 years. Human bone marrow aspiration and collection were conducted by the Orthopedic Surgery Department at the Xiangya Hospital of Central South University. Prior to the study, all participants underwent a thorough screening process which included a detailed questionnaire, medical history review, and physical examination. Those with conditions that could affect bone metabolism, such as kidney or liver diseases, parathyroid or thyroid disorders, diabetes mellitus, hyperprolactinemia, oophorectomy, rheumatoid arthritis, ankylosing spondylitis, malabsorption syndromes, malignant tumors, hematological diseases, or previous pathological fractures within the past year were excluded from the study. Participants who had received treatment with glucocorticoids, estrogens, thyroid hormone, parathyroid hormone, fluoride, bisphosphonate, calcitonin, thiazide diuretics, barbiturates, or antiseizure medication were also excluded. The bone marrow aspiration and collection were performed during bone fracture surgery for the remaining participants.

### Micro-CT analysis

The femur of mice were dissected, fixed for 24 h with 4% paraformaldehyde, and scanned using high-resolution micro-computed tomography (mCT) (Skyscan 1172, Bruker MicroCT, Kontich, Belgium).^4,5,70^ The parameters of trabecular bone in the metaphysis and cortical bone in the mid-diaphysis were analyzed using NRecon image reconstruction software version 1.6 (Bruker MicroCT), CTAn data-analysis software version 1.9 (Bruker MicroCT), and CTVol 3-dimensional model visualization software version 2.0 (Bruker MicroCT). The scanner settings were 50 kVp, 201 mA, and a resolution of 12.64 mm/pixel.

The analysis was performed as described previously.^5^ For the distal femur, the region of interest (ROI) analyzed was 5% of the femoral length, ranging from 0.1 mm below the growth plate, to determine Tb.BV/TV, Tb.N, Tb.Sp, Tb.Th. For cortical bone, cross sectional images of the mid-diaphysis of femur were used to perform 3-dimensional histomorphometric analysis of cortical bone. The ROI of cortical bone selected for analysis was of 10% of femoral length in mid-diaphysis of the femur to determine cortical thickness (Ct. Th).

### β-gal staining

For cell senescence assay, BMSCs and bone marrow adipocytes were fixed and stained by a senescence β-galactosidase staining Kit (Cell Signaling Technology, 9860), according to the manufacturer’s instructions.

For femoral bones histological analysis, femoral bones were dissected and freshly fixed in 4% paraformaldehyde overnight, followed by a 14-day decalcification in 0.5 mol/L EDTA (pH 7.4). The samples were then dehydrated in 20% sucrose plus 2% polyvinylpyrrolidone solution for 24 h and embedded in OCT. Ten mm-thick coronal sections of the femurs were obtained for SA β-gal staining according to the manufacturer’s instructions.

### Immunoprecipitation and co-immunoprecipitation

The membrane protein of bone marrow adipocyte was extracted using the Membrane and Cytosol Protein Extraction Kit (Beyotime, P0033) and were incubated with his labeled rPCLAF protein and his antibody overnight at 4 °C. Protein A/G Magnetic Beads (MedChemExpress, HY-K0202-1) was rinsed for three times and was then add into protein lysate mixture for another incubation for 2 h at room temperature. The resulting immunoprecipitants were separated using SDS-PAGE and then analyzed using MS.

To perform the immunoprecipitation, HEK293T cells were transfected with Flag-Adgrl2 plasmids and His-Pclaf plasmids respectively. The cell lysate was collected and immunoprecipitated using antibodies against His (TA150088, 1:2 000, 1:2 000, Origene) and Flag (TA50011-100, 1:100, Origene) respectively, followed by adsorption to protein G Sepharose. The resulting immunoprecipitants were separated using SDS-PAGE and blotted onto a PVDF (Millipore) membrane. The membrane was then incubated with antibodies against Flag (TA50011-100, 1:2 000, Origene) and His (TA150088, 1:2 000, Origene) respectively, and visualized using a chemiluminescence reagent (Thermo Fisher Scientific, 32106) and imaged using a ChemiDoc XRS Plus luminescent image analyzer (Bio-Rad Laboratories, USA).

### Single-cell RNA sequencing analysis

Previous published scRNA-seq data(GEO: GSE202710 and GSE137869)^5,24^ were re-analyzed here and the Cell Ranger software pipeline (version 5.0.0) provided by 10x Genomics was used to demultiplex cellular barcodes, map reads to the genome and transcriptome using the STAR aligner, and down-sample reads as required to generate normalized aggregate data across samples, producing a matrix of gene counts versus cells. Raw reads were processed with fastQC and fastp to remove low quality reads. Poly-A tails and adaptor sequences were removed by cutadapt. After quality control, reads were mapped to the reference genome Rnor_6.0 using STAR. Gene counts and UMI counts were acquired by featureCounts software. Expression matrix files for subsequent analyses were generated based on gene counts and UMI counts (Singleron Biotechnologies, Nanjing, China). (2) Quality control, dimension-reduction and clustering. Cells were filtered by gene counts below 500. Cells with over 10%itochondrial content were removed. After filtering, 23 736 cells were retained for the downstream analyses. We used functions from Seurat V3.1.2 (Satija et al. 2015) for dimension-reduction and clustering. All gene expression was normalized and scaled using NormalizeData and ScaleData. Top 2 000 variable genes were selected by FindVariableFeautres for PCA analysisWe processed the unique molecular identifier (UMI) count matrix using the R package Seurat (version 3.1.1). violin plots displaying the expression of PCLAF were generated by Seurat V3.1.2 DotPlot/Vlnplot.

In previous raw data processing (GEO: GSE202710),^24^ we scored each cluster by the normalized expressions of the following canonical markers: Neutrophils (Lcn2, Camp, Retnlg, Csf3r), Granulocytes macrophages progenitor cells (GMPs) (Elane, Mpo, Prtn3), Macrophages (S100a4, Ccl9, Cd300e, Cd68), Dendritic cells (Cd74, Irf8), Plasma cells (Cd79b, Jchain, Mzb1), B cells (Cd79b, Cd19), Late pro-B cell (Dntt, Rag1, Cd79b, Cd19), Pro-B cells (Cd19, Cd79b, Ezh2), Erythroblast (Hba-a1,Hbb-bs), T cells/NK (Cd3d, Cd8a, Nkg7), Basophils (Ms4a2, Cd63), MSCs(Lepr, Cxcl12, Kitl, Angpt1), MPPs(CD34hi, CD45hi, CD38lo, CD224hi).

In raw data processing(GEO: GSE137869),^5^ to assign one of the 13 cell types to each cluster, we scored each cluster by the normalized expressions of the following canonical markers: Neutrophils (Lcn2, Camp, Retnlg, Csf3r), Pro-neutrophils (Pglyrp1, Lcn2, Camp, Mki67), Granulocytes macrophages progenitor cells (GMPs) (Elane, Mpo, Prtn3), Macrophages (Lyz2,Cd68), Plasma cells (Cd79b, Jchain, Mzb1), B cells (Cd79b, Cd19), Late pro-B cell (Dntt, Rag1, Cd79b, Cd19), Pro-B cells (Cd19, Cd79b, Ezh2), Proerythroblast (Hbb, Tfrc, Mki67), Erythroblast (Hbb, Tfrc), T cells (Cd3d, Cd8a, Nkg7), Basophils (Ms4a2, Cd63), Megakaryocytes (Pf4). Within Macrophages, we used the following markers for subtype identification: Cluster 1 (Vcan, Slpi, Chi3l1), Cluster 2 (Ccne2, Elane, Nkg7, Ctsg), Cluster 3 (Map2k3, Lgals1, S100a9, S100a8), Cluster 4 (P2ry10, Cd74, RT1-Da, Gngt2), Cluster 5 (Top2a, Ube2c, Histlh2an, H2afx).

### Mass spectrometry analysis

For mass spectrometry analysis, membrane protein of bone marrow adipocytes extraction was carried out as previously described in ref.^16^. After digestion, samples were desalted using C18 cartridges (Empore SPE Cartridges C18 (standard density), bed I.D. 7 mm, volume 3 mL, Sigma), concentrated using vacuum centrifugation, and reconstituted in 40 mL of 0.1% (v/v) formic acid. TMT reagent was used to label 100 mg of peptide mixture from each sample, according to the manufacturer’s instructions (Thermo Scientific). LC-MS/MS analysis was performed using a Q Exactive mass spectrometer (Thermo Scientific) coupled to Easy nLC (Proxeon Biosystems, now Thermo Fisher Scientific) for 90 min. The peptides were loaded onto a reverse phase trap column (Thermo Scientific Acclaim PepMap100, 100 mm × 2 cm, nanoViper C18) connected to the C18-reversed phase analytical column (Thermo Scientific Easy Column, 10 cm long, 75 mm inner diameter, 3 mm resin) in buffer A (0.1% Formic acid) and separated with a linear gradient of buffer B (84% acetonitrile and 0.1% Formic acid) at a flow rate of 300 nL/min controlled by IntelliFlow technology. The mass spectrometer was operated in positive ion mode. MS data was acquired using a data-dependent top 10 method that dynamically chose the most abundant precursor ions from the survey scan (300–1 800 m/z) for HCD fragmentation. AGC target was set to 3e6, and maximum inject time to 10 ms. Dynamic exclusion duration was 40.0 s. Survey scans were acquired at a resolution of 70 000 at m/z 200, and resolution for HCD spectra was set to 17 500 at m/z 200, and isolation width was 2 m/z. Normalized collision energy was 30 eV, and the underfill ratio was defined as 0.1%. Peptide recognition mode was enabled. The MS raw data for each sample were searched using the MASCOT engine (Matrix Science, London, UK; version 2.2) embedded into Proteome Discoverer 1.4 software for identification and quantitation analysis. Significance was assessed with t tests. The differentially expressed peptides were subsequently filtered for median fold-change > 1.3 and *P*-value < 0.05 (Student’s t test). The mass spectrometry proteomics data will be deposited in a public repository upon acceptance.

### RNA-sequencing

RNAs were extracted from bone marrow adipocytes from adipogenic differentiation derived BMSCs which treated with rPCLAF or PBS using RNeasy Mini Kit (QIAGEN, 74014) following manufacturer’s instruction. The index-coded samples were clustered using the HiSeq PE Cluster Kit v4-cBot-HS (Illumina) on a cBot cluster generation system following the manufacturer’s instructions. Subsequently, the libraries were sequenced on an Illumina platform, and 150 bp paired-end reads were produced. The accession number for the RNA sequencing data will be deposited in a public repository upon acceptance.

Sample demultiplexing and conversion to FASTQ files was performed using Illumina’s fastq software with all default options. Reads from FASTQ files of the cultured cells were aligned to mouse genome using the STAR aligner (v2.4.2). Uniquely mapped reads were used for gene expression estimates as transcripts per million reads (TPMs). Kruskal–Wallis tests were used for identifying genes differentially expressed among sample groups. Pathway analysis was performed for functional annotation of the ASIGs in the dataset using established tools available online (EnrichR). Heatmap was formed using established online tool.

### Histological analysis

Femurs were dissected and fixed overnight with 10% paraformaldehyde at 4 °C. The samples were then decalcified using 10% EDTA (pH 7.4) for 21 days at 4 °C.

For frozen section, the samples were dehydrated in a solution consisting of 20% sucrose and 2% polyvinylpyrrolidone for a duration of 24 h and then embedded in OCT. Coronal sections of the femurs, with a thickness of ten micrometers, were obtained for SA β-gal staining by utilizing staining kit from Cell Signaling Technology (Danvers, MA). The oil O red staining was also performed according to the manufacturer’s instructions.

For paraffin sections, the samples were then embedded in paraffin. Paraffin sections with a thickness of 6 μm were prepared and stained with TRAP (Sigma-Aldrich) to quantify the number and surface of osteoclasts. For immunohistochemical staining, bone sections were treated with 0.05% trypsin at 37 °C for 15 min for antigen retrieval, followed by overnight incubation with a primary antibody against Osteocalcin (Takara, M173) at 4 °C. The HRP-streptavidin detection system (Dako) was used to detect immunoactivity, followed by counterstaining with hematoxylin (Sigma). Bone sections were incubated overnight with primary antibodies: F4/80 (123110, 1:200, BioLegend), perilipin (9349, 1:200, Cell Signaling Technology), PCLAF (sc-390515, 1:200, Santa Cruz), γH2AX (9718, 1:200, Cell Signaling Technology), p21 (sc-166630, 1:50, Santa Cruz), Osteocalcin (M173, 1:200, Takara), Leptin (BAF497, 1:200, R&D system), Ctsk (57056, 1:300, Cell Signaling Technology). Fluorescence-conjugated secondary antibodies (Jackson- ImmunoResearch, 1:200) were used to detect fluorescent signals.

### Calcein double-labeling

Mice were intraperitoneally injected with calcein (10 mg/kg, Sigma) 10 days and 3 days before euthanasia. After separation, the femur was immersed in 70% ethanol and embedded in methyl methacrylate. The samples were cut into 5 mm with a hard tissue cutter and examined under a fluorescence microscope to evaluate the mineral attachment rate using Image-Pro Plus 6.0. MAR is the distance between two labels divided by the time between labels.

### Western blot analysis

Western blot analysis was conducted as previously described in refs. ^24,71^. Briefly, cells were lysed in RIPA lysis buffer and harvested with a rubber policeman. Samples (20 mg protein) were loaded into pre-cast electrophoresis gels (Bio-Rad), separated by SDS-PAGE and electro-transferred onto a PVDF membrane. After blocking with 5% non-fat milk, the blots were incubated with primary antibodies overnight at 4 °C and then with secondary antibodies at room temperature for 1 h.

The primary antibodies used were AKT (4685, 1:1 000, Cell Signaling Technology), phospho-AKT (4060, 1:1 000, Cell Signaling Technology), PCLAF (sc-390515, 1:500, Santa Cruz), mTOR (2983, 1:1 000, Cell Signaling Technology), phospho-mTOR (5536, 1:1 000, Abcam), ADGRL2 (140830, 1:1 000, Abcam). The protein bands were visualized using a chemiluminescence reagent (Thermo Fisher Scientific, 32106).

### RNA isolation and qRT-PCR analysis

Total RNA was extracted using TRIzol (Sigma, T9424) and converted into cDNA using the PrimeScript RT Reagent Kit (Takara, PR037A). RNA of BMAds was extracted using Total RNA kits (Omega, R1034) according to the manufacturer’s instructions. Real-time reverse transcription PCR was performed using the ABI QuantStudio 3 system. Amplification reactions were prepared in 25 μL reaction volumes with SYBR Green, cDNA, and amplification primers. The primer sequences used are listed in Table S1.

### PCLAF neutralizing antibody

Five male Balb/c mice (2-3 months, each weighing about 16–20 g) were immunized with a dose of 50 μg/mouse with the immunogen PCLAF (MVRTKADSVPGTYRKVVAARAPRKVLGSSTSATNSTSVSSRKAENKYAGGNPVCVRPTPKWQKGIGEFFRLSPKDSEKENQIPEEAGSSGLGKAKRKACPLQPDHTNDEKE) mixed with an equal volume of adjuvant (complete/incomplete Freund’s adjuvant). The adjuvant was purchased from BD Company (263910). After mixed together, they were injected subcutaneously at multiple points in the abdomen, with the second immunization at an interval of 2 weeks and the third immunization at another interval of 3 weeks. After 3 days of enhanced immunization, the spleen was taken for hybridoma fusion. One week after the last immunization, 50–60 mL of blood was drawn from the orbital vein clusters of mice. After standing at 4 °C overnight, the upper serum was separated by centrifugation for detection. A suitable coating buffer was used to dilute the amount of the detected protein to 5 mg/mL, and then 100 mL was added to each well of the 96-well plate and coated overnight at 4 °C. The plate was washed once with washing buffer at 200 mL/well. Then it was sealed with a 300 mL/hole sealing plate and buffered for 1 h at room temperature. The plate was washed twice with washing buffer, and then the sample (gradient diluted sample and sample diluent 100 mL/well) was added, and the antibody (100 mL/well to 96-well plate) was detected at room temperature for 2 h. Wash the plate and add the reaction buffer to 200 mL/ well, and place at room temperature for 12 min. Finally, 50 mL/well termination buffer was added to stop the reaction, and the microplate analyzer with a detection wavelength of 450 nm was used for detection. All spleen cells of immunized mice were mixed with mouse myeloma cells at a ratio of 1:1, and hybrid cells were obtained by electrofusion. The antigen protein was coated, and the cell supernatant was determined by ELISA. The positive wells were selected and cloned by dilution until the hybridoma cell line stably obtained the secreted monoclonal antibody. After filtration, a total of 6 hybridoma cells were obtained. Subsequently, the hybridoma cell clone numbered MM01 was selected for antibody preparation. The 1 mL hybridoma cells were transferred to a 100 mL culture flask, and a certain amount of medium was added regularly for cell amplification. The cells were cultured for 10–12 days. The protein A affinity chromatography column was washed with ultrapure water, and then the equilibrium buffer was balanced. The supernatant of the treated hybridoma cells was loaded onto an affinity chromatography column. After loading, the cells were washed with equilibrium buffer. The elution buffer was eluted and the elution peak was collected. After neutralization, Tris buffer was used to desalt to PBS7.4 to obtain PCLAF-Nab for in vivo and in vitro experiments.

### Quantification and statistical analysis

The GraphPad Prism 8 software (GraphPad Software) was used for statistical analysis. The datas downloaded from GTEx program were analized with R version 4.0.2. Cell-based experiments were repeated at least twice, while at least three mice were used for each group in animal experiments unless otherwise specified. Data is presented as mean ± SD. Data showed a continuous normal distribution. Two-tailed Student’s *t* test was used for comparisons between two groups, and one- or two- way ANOVA were used for comparisons among multiple groups. Pearson’s Correlation analysis was used for correlation coefficient. Statistically significant differences were denoted as follows: * represents *P* < 0.05, ** represents *P* < 0.01, *** represents *P* < 0.001.

### Supplementary information

supplemental material

## Data Availability

Previous published scRNA-seq data were re-analyzed here and the accession number was GSE137869 and GSE202710. Previous published mass spectrometry proteomics data was re-analyzed here which was deposited to the ProteomeXchange Consortium via the PRIDE: PXD027548.^72^ The RNA sequencing data will be deposited in a public repository upon acceptance. The expression data of PCLAF and senescence-associated secretory phenotype genes was derived from the GTEx program (https://www.gtexportal.org/). All data and material utilized for this paper are available upon requests to the corresponding author.
