# Supplementary material for: PCLAF induces bone marrow adipocyte senescence and contributes to skeletal aging
Source: Bone Res. 2024 Jul 4;12:38. doi: 10.1038/s41413-024-00337-5 (PMC11222446; doi:10.1038/s41413-024-00337-5)
Supplement: Supplementary file 1 — supplemental material [file 41413_2024_337_MOESM1_ESM.docx]

**SUPPLEMENTAL MATERIAL FOR**

**PCLAF induces bone marrow adipocyte senescence and contributes to skeletal aging**

Ling-qi Xie^1,^**^#^**, Ya-lun Cheng^1,^**^#^**, Biao Hu^1^, Xin Chen^1^, Yu-ze An^1^, Zhu-ying Xia^1^, Guang-ping Cai^1^, Chang-jun Li^1.2.3^, Hui Peng^1,*^

**Fig. S1**
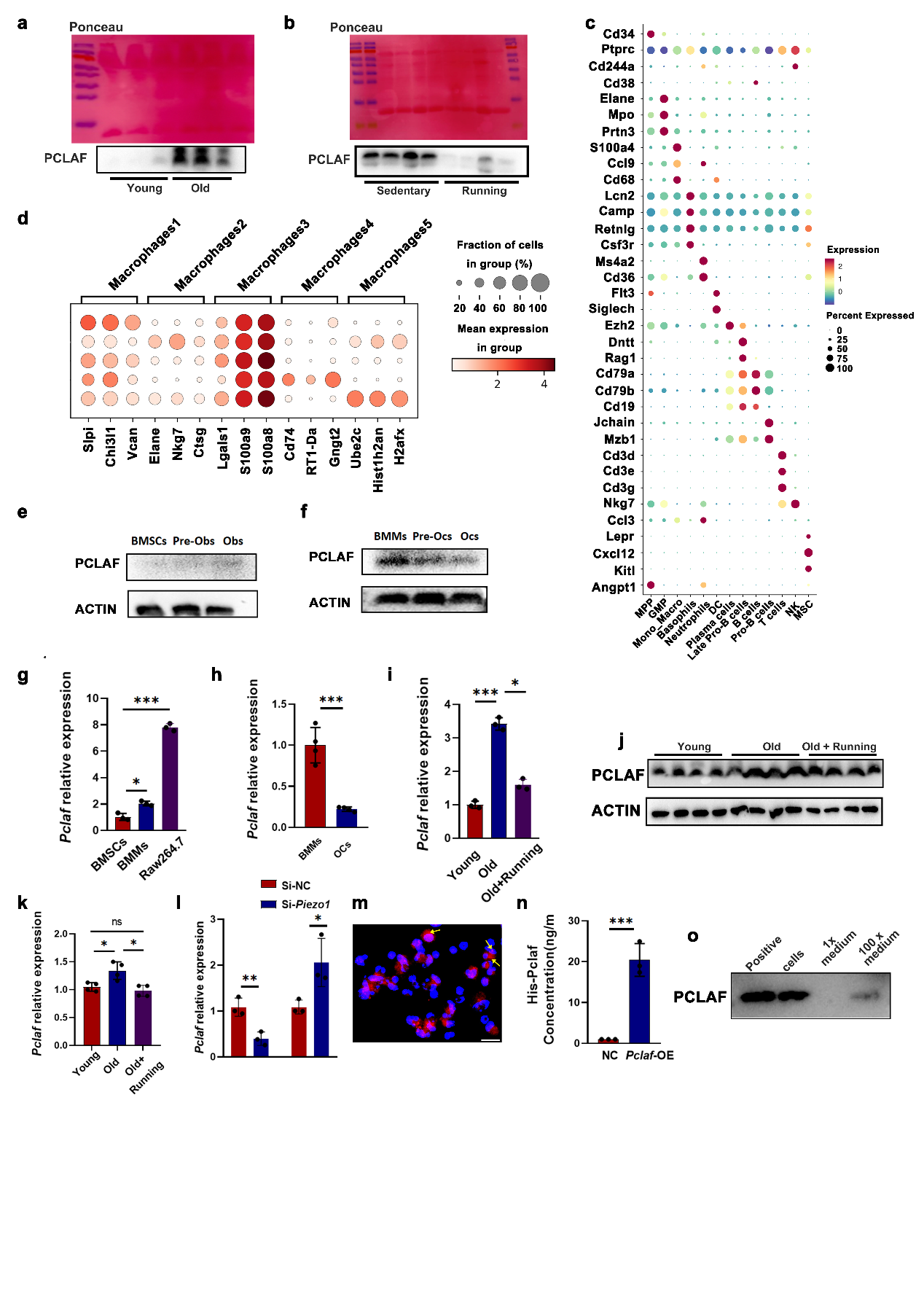


**Fig. S1 (related to Fig 2) PCLAF is abundant in bone marrow macrophages.**

**a, b** Representative Ponceau S staining and western blot images of PCLAF expression in bone marrow supernatant from young versus old (**a**) and sedentary versus running mcie (**b**).

**c** A dot plot demonstrating defining genes for each cell cluster in the bone marrow (ground versus running mice).

**d** A dot plot demonstrating defining genes for five clusters of bone marrow macrophages (young versus old rats).

**e** Western blot images of PCLAF expression in BMSCs, pre-osteoblasts (Pre-Obs) and osteoblasts (Obs).

**f** Western blot images of PCLAF expression in bone marrow macrophages (BMMs), pre-osteoclasts (Pre-Ocs) and osteoclasts (Ocs),

**g, h** QPCR analysis of *Pclaf* expression in BMSCs, BMMs, Raw264.7 and Ocs.

**i** QPCR analysis of *Pclaf* expression in BMMs from 3-month-old mice, 15-month-old running mice and sedentary mice (n=4).

**j, k** Western blot image (**j**) and quantification analysis (**k**) of PCLAF expression in BMMs from 3-month-old mice, 15-month-old running mice and sedentary mice (n=4).

**l** QPCR analysis of *Pclaf* and *piezo1* expression in RAW264.7 transfected with siRNA-*Piezo1* or siRNA-*NC* (n = 3).

**m** Representative images of immunofluorescence staining of PCLAF (yellow arrows) in cells (scale bars, 50 um).

**n** Quantitative analysis of His-PCLAF concentration in culture medium of HEK293T cells transfected with His-*Pclaf* plasmid and control plasmid.

**o** Representative western blot image of PCLAF expression in macrophages lystate and concentrated culture medium.

Data are shown as the mean ± SD. *p < 0.05, **p < 0.01, ***p < 0.001 by Student’s *t*-test (**h, l, n**) or by two-way ANOVA (**g, i, k**).﻿

**Fig. S2**

**
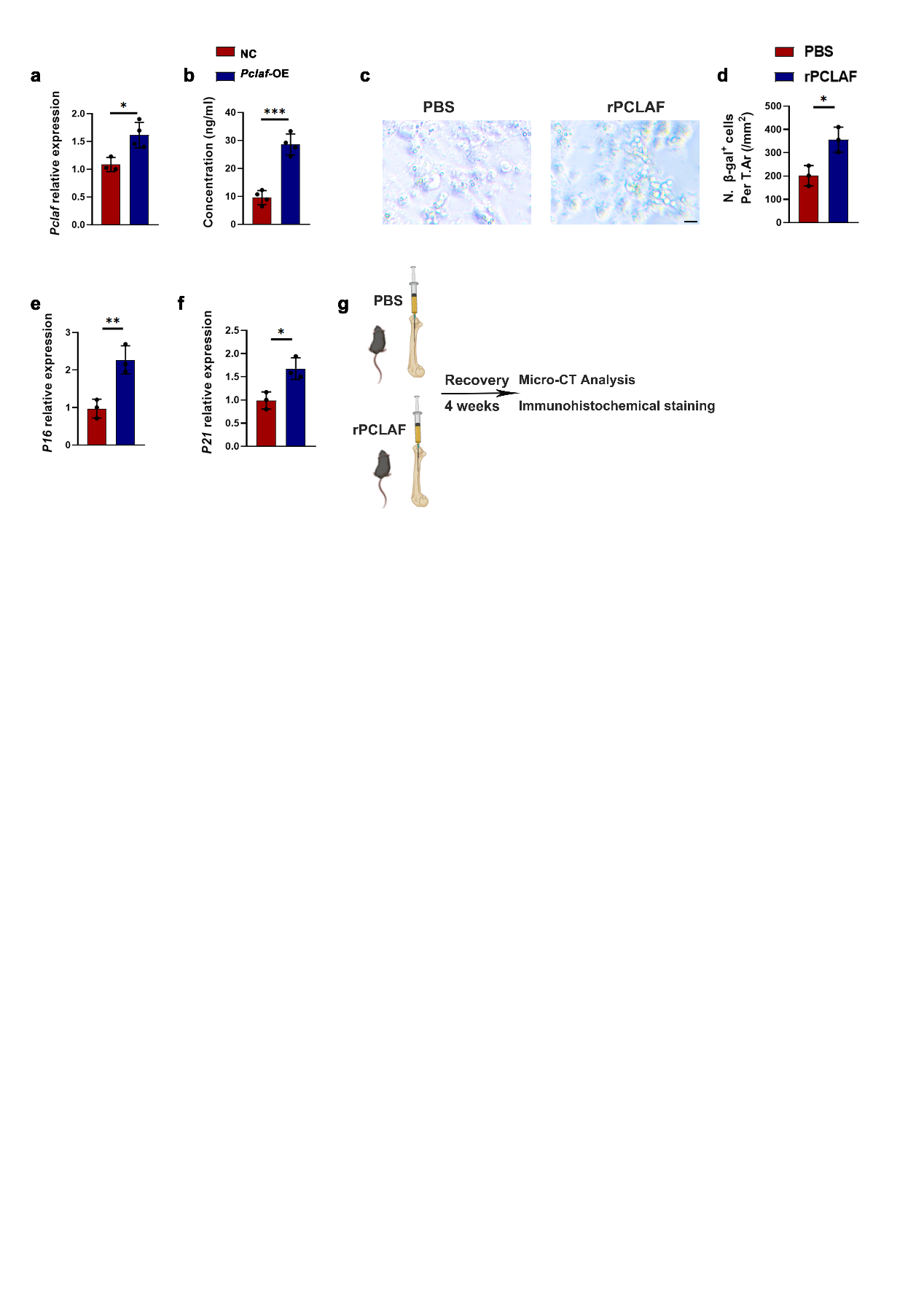
**

**Fig. S2 (related to Fig 3) PCLAF induces BMAds senescence in vitro.**

**a** QPCR analysis of *Pclaf* expression in BMMs transfected with plasmid-*Pclaf* (*Pclaf*-OE) or control plasmid (NC) (n=3).

**b** Quantitative analysis of PCLAF in the conditional medium (n=4).

**c, d** Representative images (**c**) and quantification (**d**) of *SA-βGal* staining of BMSCs-derived adipocytes treated with rPCLAF or PBS (scale bar, 50um; n = 3).

**e, f** QPCR analysis of *p16* (**e**) and *p21* (**f**) expression of BMSCs-derived adipocytes treated with rPCLAF or PBS.

**g** Pattern diagram of reproduction strategy for mice intra-femorally injected with PBS or rPCLAF.

Data are shown as the mean ± SD. *p < 0.05, **p < 0.01, ***p < 0.001 by Student’s *t*-test (**a, b, d-f**).

**Fig. S3**

**
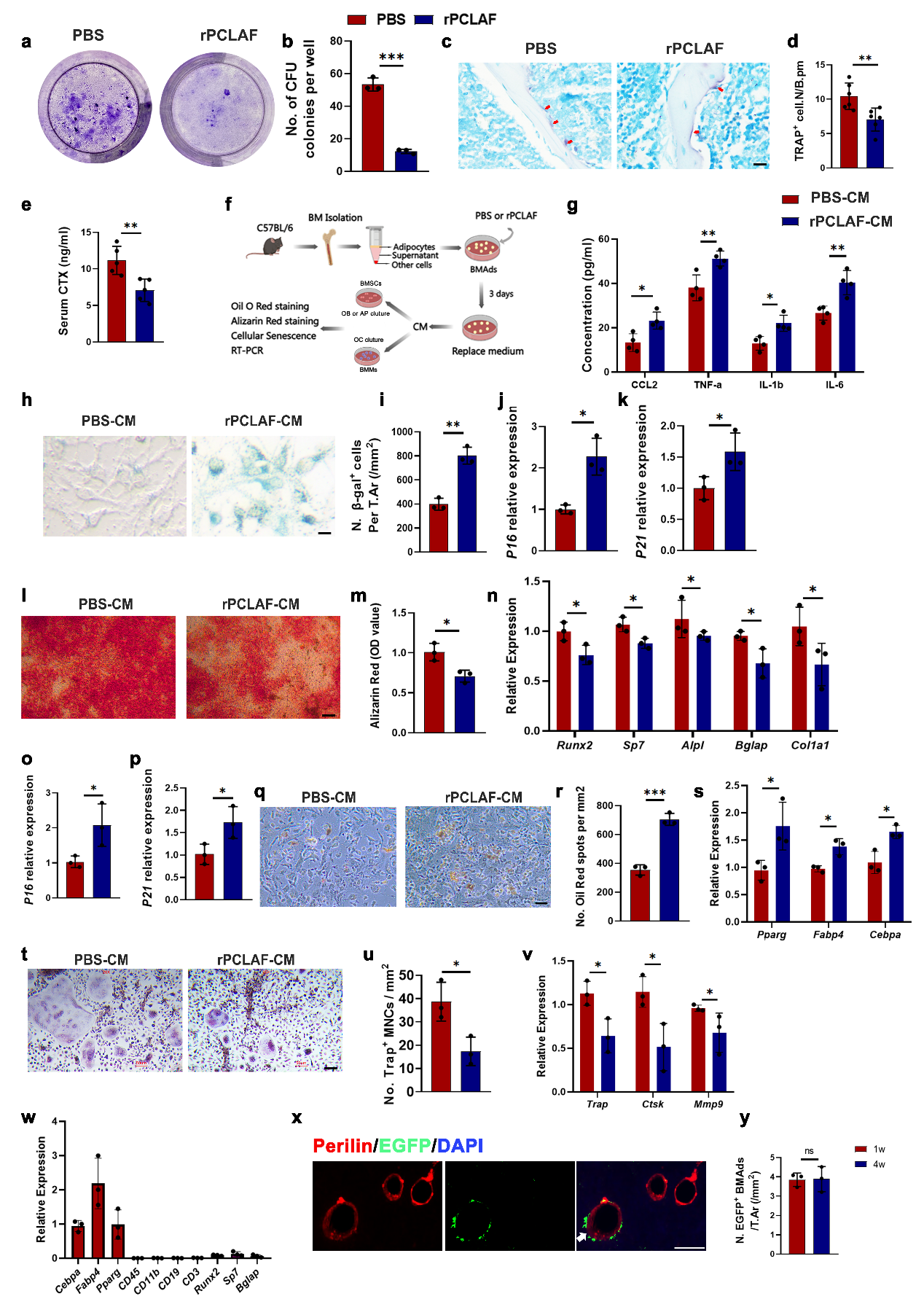
**

**Fig. S3 (related to Fig 4) Senescent BMAds impaire osteogenesis and osteoclastogenesis .**

**a, b** Representative images (**a**) and quantification analysis (**b**) of ﻿colony-forming unit (CFU) of BMSCs (n=3).

**c, d** Representative images of TRAP staining (**c**) and quantification (**d**) of number of TRAP^+^ cells (red arrows) in femurs (scale bar, 50um; n = 6).

**e** Serum CTX of PBS and rPCLAF-treated mice (n = 6).

**f** Pattern diagram of preparation of the conditioned medium (CM) of cultured BMAds and intervention for BMSCs or BMMs.

**g** Quantitative analysis of SASPs in the CM of BMAds (n=4).

**h, i** Representative images (**h**) and quantification analysis (**i**) of *β-gal* staining of BMSCs (scale bar, 50um; n = 3).

**j, k** QPCR analysis of *p16* (**j**) and *p21* (**k**) of BMSCs (n=3).

**l, m** Representative images of Alizarin Red staining (**l**) and quantification (**m**) of staining (scale bar, 50um; n = 3).

**n** QPCR analysis of osteogenic makers of BMSCs-differentiated osteoblasts, including *Runx2*, *Sp7*, *Alpl, Bglap* and *Col1a1*(n=3).

**o, p** QPCR analysis of *p16* (**o**) and *p21* (**p**) of BMSCs- differentiated osteoblasts (n=3).

**q, r** Representative images of Oil Red staining (**q**) and quantification (**r**) of staining (scale bar, 50um; n = 3).

**s** QPCR analysis of adipogenic makers of BMSCs, including *Pparg*, *Fabp4* and *Cebpa* (n=3).

**t, u** Representative images of TRAP staining (**t**) and quantification (**u**) of staining of osteoclasts (scale bar, 50um; n = 3).

**v** QPCR analysis of osteoclastic makers of BMMs, including *Ctsk*, *Trap* and *Mmp9* (n=3).

**w** QPCR analysis of adipogenic, osteogenic and immune-related genes of BMAds (n=3).

**x, y** Representative images (x) and quantification (y) of immunofluorescence staining of EGFP and perilipin (scale bar, 50um; n = 3).

Data are shown as the mean ± SD. *p < 0.05, **p < 0.01, ***p < 0.001, by Student’s *t*- test (**b, d, e, g, i-k, m-p, r-s, u-v, y**).

**Fig. S4**

**
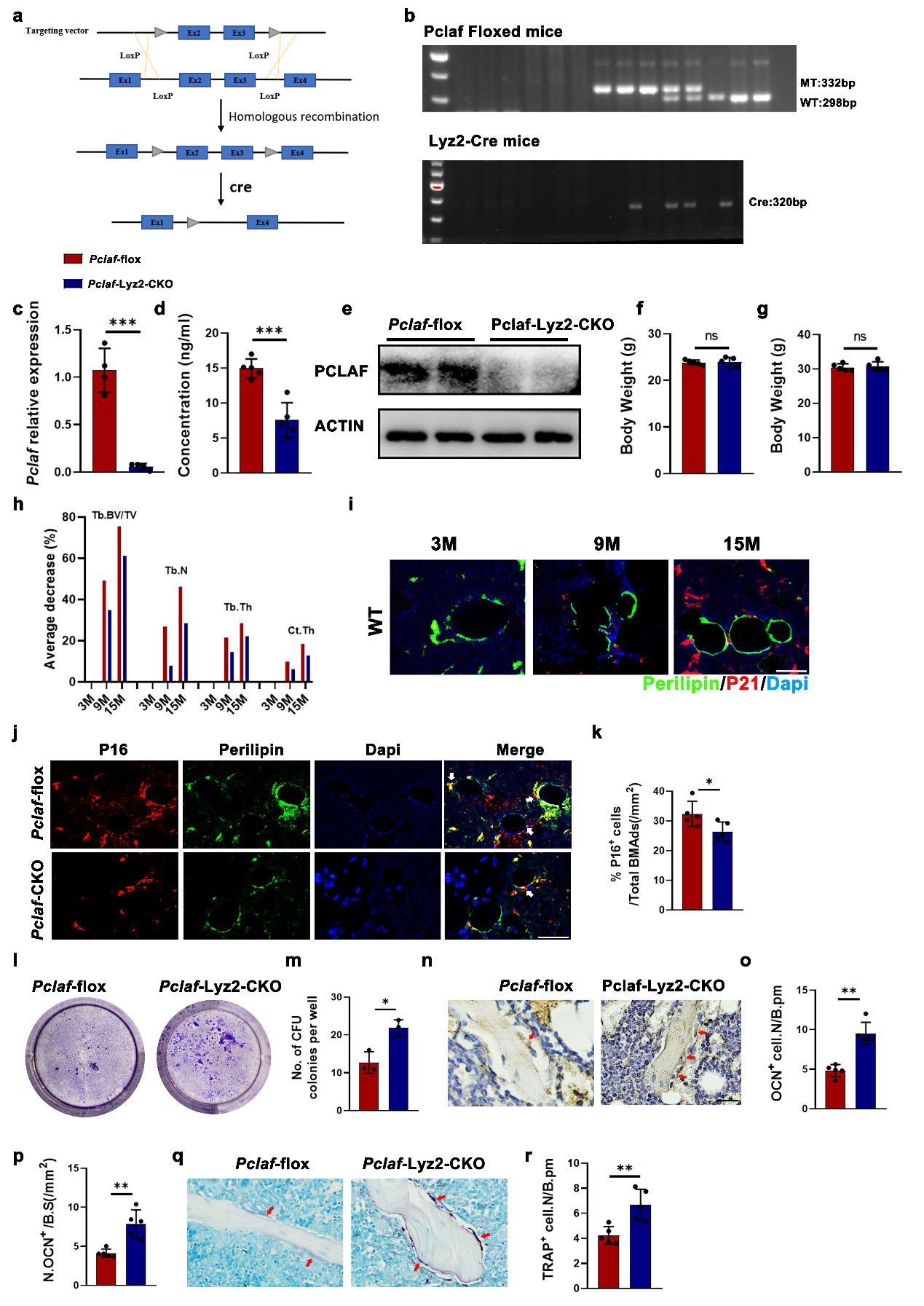
**

**Fig. S4 (related to Fig 5) The deficiency of *Pclaf* in vivo promotes bone formation.**

**a** Pattern diagram of the construction of *Pclaf*-Lyz2-CKO mice.

**b** PCR images of genotyping datas of *Pclaf-*floxed mice and *Lyz2-Cre* mice.

**c, d** QPCR analysis of *Pclaf* expression in BMMs (**c**) and quantitative analysis of PCLAF (**d**) in the bone marrow supernatants of *Pclaf*-Lyz2-CKO and *Pclaf*^flox^ mice (n=5).

**e** Western blot analysis of PCLAF in BMMs.

**f, g** Body weight of 9-months old (**f**) and 15-months old (**g**) *Pclaf*-Lyz2-CKO mice and *Pclaf*^flox^ mice (n=5).

**h** The average decreased rate of bone paremeters of 3-, 9-, 15-month-old *Pclaf*-Lyz2-CKO mice and *Pclaf*^flox^ mice.

**i** Representative images of co-localization staining of P21 and perilipin of femurs from 3-, 9-, 15-month-old WT mice (scale bar, 50um; n = 5).

**j, k** Representative images of co-localization staining (**j**) of P16 and perilipin (scale bar, 50um; n = 4) and quantification (**k**) of the percentage of P16^+^ cells in total perilipin^+^ cell population (% P16^+^ cells/total BMAds) (n = 5).

**l, m** Representative images (**l**) and quantification analysis (**m**) of ﻿colony-forming unit (CFU) of BMSCs of *Pclaf*-Lyz2-CKO and *Pclaf*^flox^ mice.

**n-p** Representative images of OCN staining (**n**) and quantification (**o, p**) of number of OCN^+^ cells (red arrows) in femurs (scale bar, 50um; n = 5).

**q, r** Representative images of TRAP staining (**q**) and quantification (**r**) of number of TRAP^+^ cells (red arrows) in femurs (scale bar, 50um; n = 5).

Data are shown as the mean ± SD. *p < 0.05, **p < 0.01, ***p < 0.001, ns, no significance by Student’s *t*-test (**c-d, f-g, k, m, o, p, r**).

**Fig. S5**

**
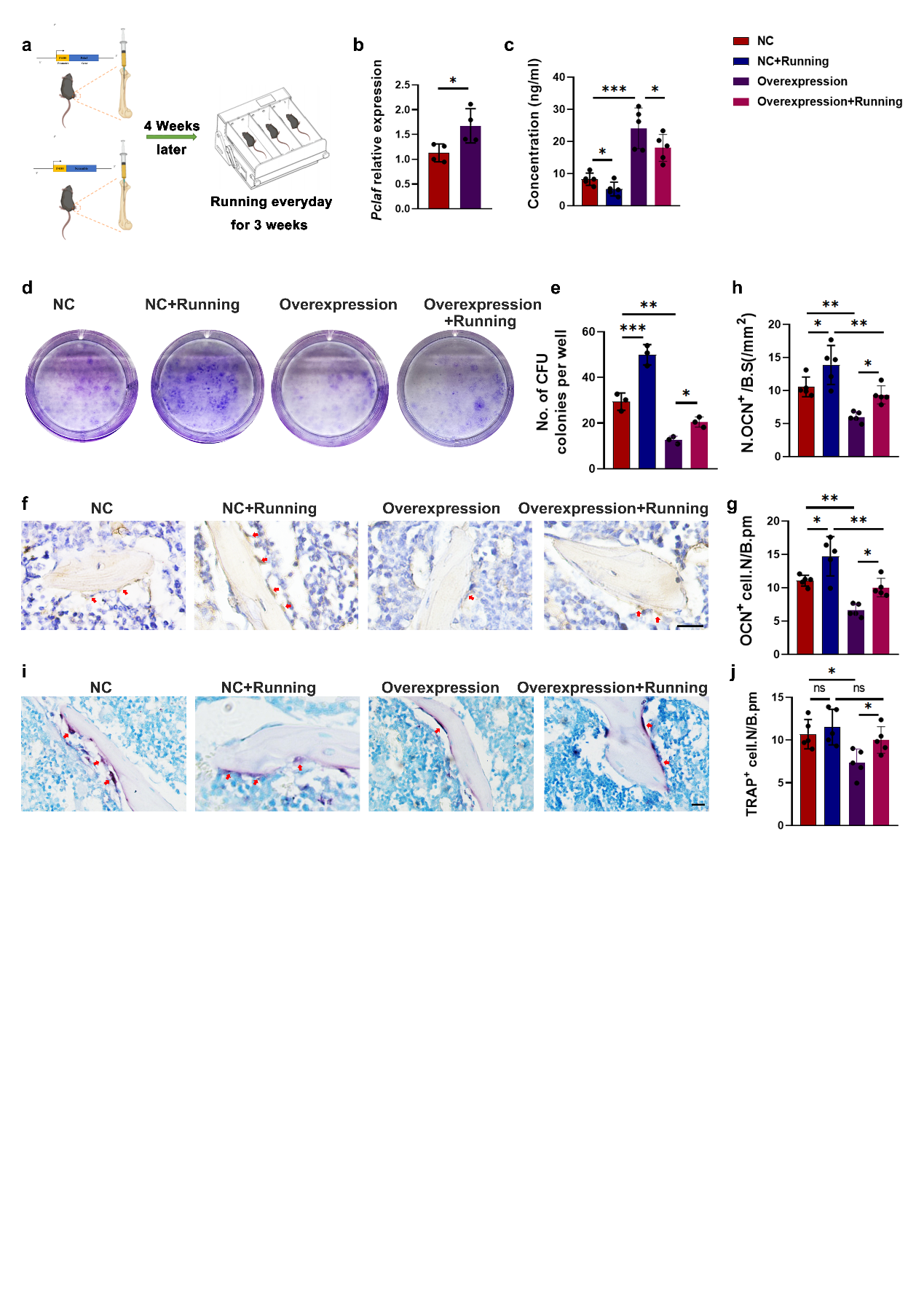
**

**Fig. S5 (related to Fig 5) Exercise alleviates PCLAF-induced bone loss.**

**a** Pattern diagram of running intervention of mice either injected AAV-F4/80-*Pclaf* or AAV-F4/80-empty.

**b** QPCR analysis of *Pclaf* expression of BMMs from the control or overexpression group mice (n=4).

**c** ELISA quantification of PCLAF in bone marrow supernatant from control and overexpression mice either undergoing running intervention (n=4).

**d, e** Representative images (**d**) and quantification analysis (**e**) of ﻿colony-forming unit (CFU) of BMSCs (n=3).

**f-h** Representative images (n=20 photographs from 4 experiments) of osteocalcin (OCN) staining (**f**) and quantification (**g, h**) of number of OCN^+^ cells (red arrows) in femurs (scale bar, 50um; n = 5).

**i, j** Representative images of TRAP staining (**i**) and quantification (**j**) of number of TRAP^+^ cells (red arrows) in femurs (scale bar, 50um; n = 5).

Data are shown as the mean ± SD. *p < 0.05, **p < 0.01, ***p < 0.001 by Student’s *t*-test (**b**) or two-way ANOVA (**c, e, g, h, j**).

**Fig. S6**

**
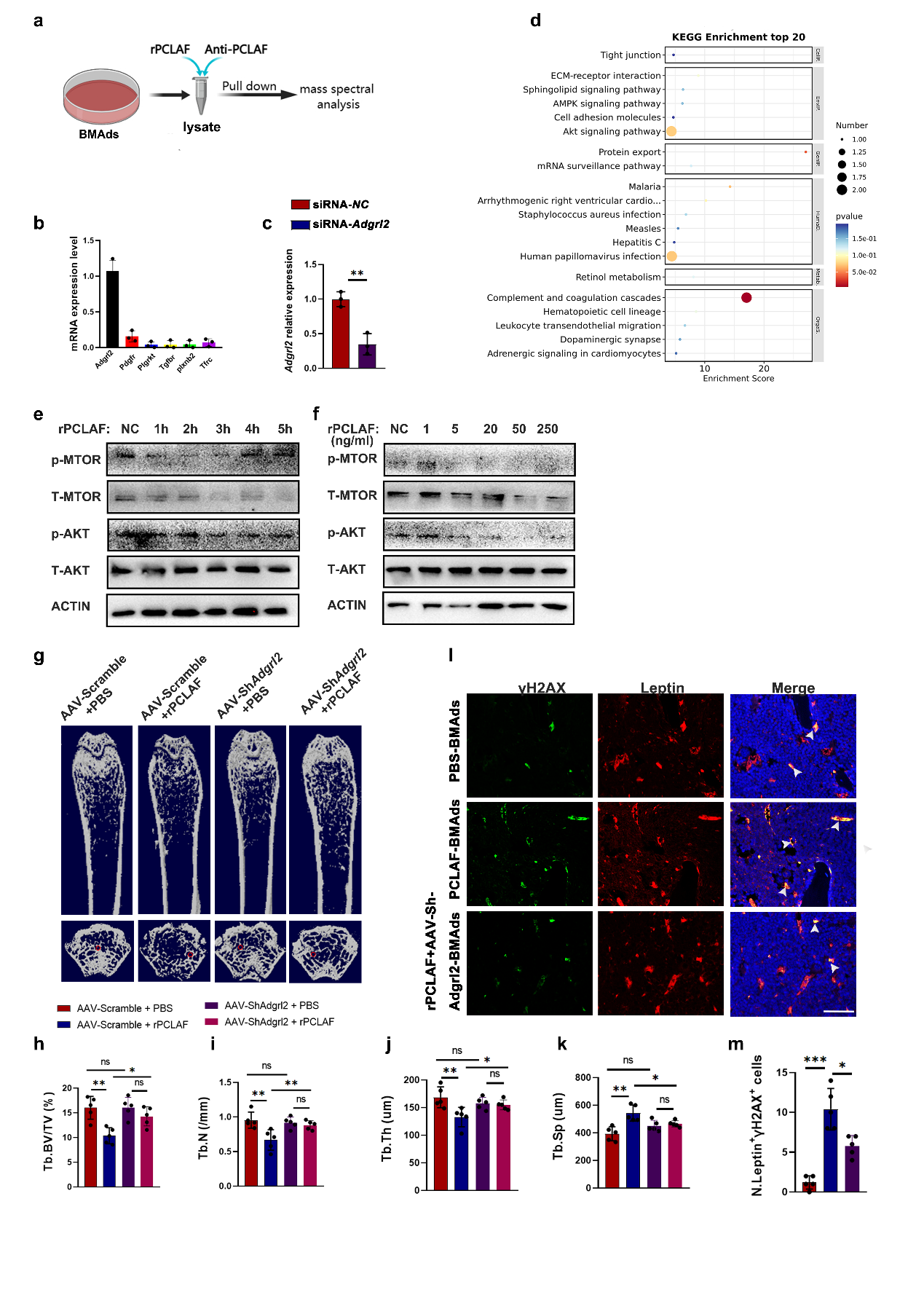
**

**Fig. S6 (related to Fig 6) PCLAF inhibits the AKT/mTOR signaling.**

**a** Pattern diagram of LC-MS/MS of lystate of BMSCs-derived adipocytes incubated with rPCLAF and PCLAF antibody.

**b** QPCR analysis of acceptors of PCLAF identified by LC-MS/MS.

**c** QPCR analysis of *Adgrl2* expression in BMSCs-derived adipocytes transfected with siRNA-*Adgrl2* or siRNA-*NC* (n=3).

**d** KEGG analysis of RNA-seq data from BMAds treated with rPCLAF.

**e, f** Representative western blot images of p-mTOR, T-mTOR, p-AKT, T-AKT in BMSCs-derived adipocytes treated with rPCLAF at indicated time (1, 2, 3, 4, 5 hours, **e**) or indicated dose (1, 5, 20, 50, 250ng/ml, **f**).

**g** Representative images of Micro-CT of mice intra-femorally injected with AAV-sh*Adgrl2* or AAV-Sramble followed by injection of PBS or rPCLAF (n=5).

**h-k** Quantitative analysis of Tb.BV/TV (**h**), Tb.N (**i**), Tb.Th (**j**), Tb,Sp (**k**) (n=5).

**l, m** Representative images of co-localization staining (**l**) of Leptin and γ-H2AX and quantification (**m**) of numbers of Leptin and γ-H2AX double-positive cells per mm^2^ tissue area (N. Leptin^+^γ-H2AX^+^ cells) (scale bar, 50um; n = 5).

Data are shown as the mean ± SD. *p < 0.05, **p < 0.01, ***p < 0.001 by Student’s *t*-test (**c**) or two-way ANOVA (**h-k, m**).

**Fig. S7**

**
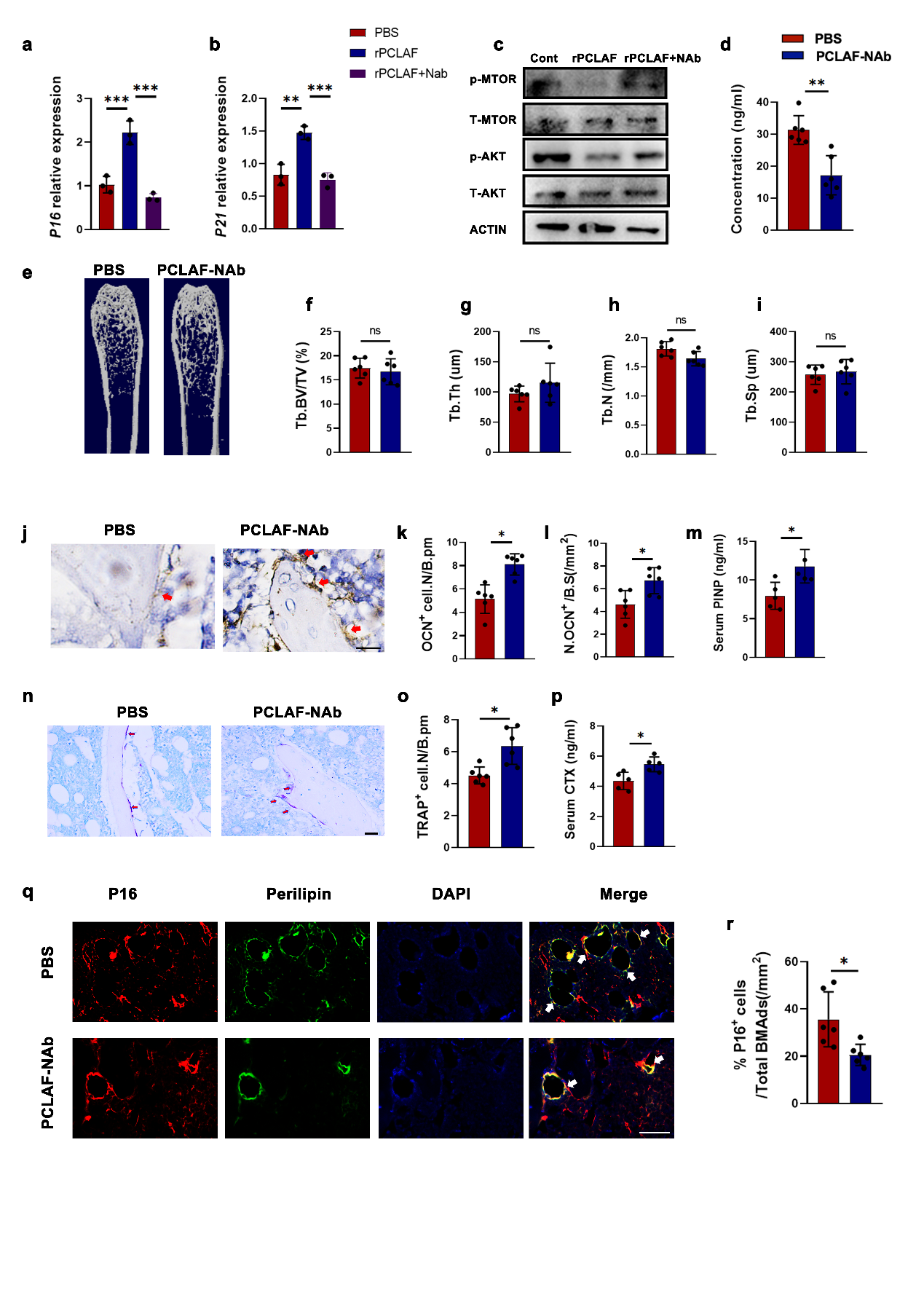
**

**Fig. S7 (related to Fig 7) PCLAF-neutralizing antibody inhibits BMAds senescence.**

**a, b** QPCR analysis of *p16* (**a**) and *p21* (**b**) of BMSCs-derived adipocytes treated withPBS, rPCLAF OR rPCLAF-Nab (n=3).

**c** Representative western blot (n = 3 in total) of AKT, mTOR protein/phosphorylation level in BMSCs-derived adipocytes.

**d** Quantification analysis of PCLAF in bone marrow supernatant from old mice treated with PBS or rPCLAF-Nab (n=6).

**e** Representative images of Micro-CT of 3-month-old mice injected with PBS or rPCLAF-Nab (n=6).

**f-i** Quantitative analysis of Tb.BV/TV (**f**), Tb.Th (**g**), Tb.N (**h**), Tb,Sp (**i**) (n=6).

**j-l** Representative images of osteocalcin (OCN) staining (**j**) and quantification (**k, l**) of number of OCN^+^ cells (red arrows) in femurs (scale bar, 50um; n = 6).

**m** Serum PINP level of PCLAF-NAb and PBS-treated mice (n=6).

**n, o** Representative images of TRAP staining (**n**) and quantification of number of TRAP^+^ cells (red arrows) in femurs (**o**) (scale bar, 50um; n = 6).

**p** Serum CTX level of PCLAF-NAb and PBS-treated mice (n=6).

**q, r** Representative images of co-localization staining (**q**) of P16 and perilipin (scale bar, 50um) and quantification (**r**) of the percentage of P16^+^ cells in total perilipin^+^ cell population (% P16^+^ cells/total BMAds) (n=6).

Data are shown as the mean ± SD. *p < 0.05, **p < 0.01, ***p < 0.01 by Student’s *t*-test (**d, f-i, k-m, o-p, r**) or two-way ANOVA (**a-b**).

**Supplementary Table 1 Nucleotide sequences of primers used for quantitative RT-PCR detection.**

| Genes  (mouse) | Forward primer (5’-3’) | Reverse primer (5’-3’) | PrimerBank ID |
| --- | --- | --- | --- |
| *p16* | GCTCAACTACGGTGCAGATTC | GCACGATGTCTTGATGTCCC | 98986443c1 |
| *Pclaf* | ACCAAAGCAAACTACGTTCCA | TTTTCCCGACGAACTTGAAGAA | 13386008a1 |
| *p21* | CCTGGTGATGTCCGACCTG | CCATGAGCGCATCGCAATC | 162287332c1 |
| *Bglap* | GCAATAAGGTAGTGAACAGACTCC | CCATAGATGCGTTTGTAGGCGG | N/A |
| *Runx2* | ATCCCCATCCATCCACTCCA | GAACTGCCTGGGGTCTGAAA | N/A |
| *Sp7* | CTCGGTTCTCTCCATCTGCC | TCTTTGTGCCTCCTTTCCCC | N/A |
| *Alpl*  *Col1a1* | AACCCAGACACAAGCATTCC  CCTCAGGGTATTGCTGGACAAC | CGGGCTCAAAGAGACCTAAG  CAGAAGGACCTTGTTTGCCAGG | N/A  N/A |
| *Pparg* | ATGGTTGACACAGAGATGC | GAATGCGAGTGGTCTTCC | N/A |
| *Cebpa* | GCGGGAACGCAACAACATC | GTCACTGGTCAACTCCAGCAC | 131886531c1 |
| *Fabp4* | AAGGTGAAGAGCATCATAACCCT | TCACGCCTTTCATAACACATTCC | 14149635a1 |
| *Adgrl2*  *Mmp9*  *Ctsk*  *Acp5* | TGACTCAAAGGTGCAACAATCG  GCAGAGGCATACTTGTACCG  GAAGAAGACTCACCAGAAGCAG  CACTCCCACCCTGAGATTTGT | TAAGTTCCGGGACATGGATCA  TGATGTTATGATGGTCCCACTTG  TCCAGGTTATGGGCAGAGATT  CCCCAGAGACATGATGAAGTCA | 124486820c3  31560795c1  N/A  N/A |
| *CD45* | CTTCAGTGGTCCCATTGTGGTG | TCAGACACCTCTGTCGCCTTAG | N/A |
| *CD11b* | TACTTCGGGCAGTCTCTGAGTG | ATGGTTGCCTCCAGTCTCAGCA | N/A |
| *CD19* | GCCACAGCTTTAGATGAAGGCAC | CATCCACCAGTTCTCAACAGCC | N/A |
| *CD3* | GCTCCAGGATTTCTCGGAAGTC | ATGGCTACTGCTGTCAGGTCCA | N/A |
